# Diguanylate Cyclase Null Mutant Reveals That C-Di-GMP Pathway Regulates the Motility and Adherence of the Extremophile Bacterium *Acidithiobacillus caldus*

**DOI:** 10.1371/journal.pone.0116399

**Published:** 2015-02-17

**Authors:** Matías Castro, Shelly M. Deane, Lina Ruiz, Douglas E. Rawlings, Nicolas Guiliani

**Affiliations:** 1 Laboratorio de Comunicación Bacteriana, Departamento de Biología, Facultad de Ciencias, Universidad de Chile, Santiago, Chile; 2 Department of Microbiology, Stellenbosch University, Stellenbosch, South Africa; Missouri University of Science and Technology, UNITED STATES

## Abstract

An understanding of biofilm formation is relevant to the design of biological strategies to improve the efficiency of the bioleaching process and to prevent environmental damages caused by acid mine/rock drainage. For this reason, our laboratory is focused on the characterization of the molecular mechanisms involved in biofilm formation in different biomining bacteria. In many bacteria, the intracellular levels of c-di-GMP molecules regulate the transition from the motile planktonic state to sessile community-based behaviors, such as biofilm development, through different kinds of effectors. Thus, we recently started a study of the c-di-GMP pathway in several biomining bacteria including *Acidithiobacillus caldus*. C-di-GMP molecules are synthesized by diguanylate cyclases (DGCs) and degraded by phosphodiesterases (PDEs). We previously reported the existence of intermediates involved in c-di-GMP pathway from different *Acidithiobacillus* species. Here, we report our work related to *At. caldus* ATCC 51756. We identified several putative-ORFs encoding DGC and PDE and effector proteins. By using total RNA extracted from *At. caldus* cells and RT-PCR, we demonstrated that these genes are expressed. We also demonstrated the presence of c-di-GMP by mass spectrometry and showed that genes for several of the DGC enzymes were functional by heterologous genetic complementation in *Salmonella enterica serovar* Typhimurium mutants. Moreover, we developed a DGC defective mutant strain (Δ*c1319*) that strongly indicated that the c-di-GMP pathway regulates the swarming motility and adherence to sulfur surfaces by *At. caldus*. Together, our results revealed that *At. caldus* possesses a functional c-di-GMP pathway which could be significant for ores colonization during the bioleaching process.

## Introduction

*Acidithiobacillus caldus* is an acidophilic, Gram-negative bacterium which gains energy by oxidation of elemental sulfur and reduced inorganic sulfur compounds [1]. Unlike others members of the *Acidithiobacillus* genus (*At*. *ferrooxidans*, *At*. *thiooxidans*, *At*. *albertensis*, *At*. *ferrivorans* and *At*. *ferridurans*), *At*. *caldus* is moderately thermophilic [1]. It has been isolated from many commercial biomining plants [2–5], together with the iron oxidizing bacteria belonging to the genus *Leptospirillum*, and dominates bacterial populations in processes operating within the temperature range of 35–50°C [2]. In bioleaching processes, ore dissolution can be achieved by two pathways, in which iron and sulfur oxidizing bacteria have specific roles [6–8]. As with other bioleaching bacteria, most *At*. *caldus* cells appear to attach to mineral surfaces [9]. In related bacterial species, such as *At*. *ferrooxidans* and *At*. *thiooxidans*, it has been shown that cell attachment to ore is determined by the presence of extracellular polymeric substances (EPS). EPS production by *At*. *ferrooxidans* has been directly correlated with bioleaching rates [10]. Even though a chemotactic response to sulfur gradients has been suggested [9], molecular mechanisms involved in the transition from planktonic to attached state by *At*. *caldus* are still unknown.

Quorum sensing (QS) and the cyclic diguanilate (c-di-GMP) pathway are the two main signalling mechanisms involved in the regulation of biofilm formation by bacteria. QS is a cell-to-cell signalling system mostly mediated by N-acyl homoserine lactones (AHL) that allows the coordination of bacterial behaviour in a cell-density-dependent manner. A functional QS system has been reported in the acidophilic bacterium *At*. *ferrooxidans* [11–12]. In this biomining bacterium, AHL-signaling molecules have been recently linked to exopolysaccharide (EPS) production and biofilm formation [13–14].

C-di-GMP is a second messenger mostly used by bacteria [15–19]. The characteristics of the phenotypes regulated by c-di-GMP signalling are still being discovered, however, it is well established that its main role is to control the switch between motile planktonic and biofilm-associated ‘lifestyles’ [20–22]. C-di-GMP is synthesized from two GTP molecules by DGCs and degraded by PDEs [23]. DGC activity is associated with GGDEF protein domains [24–25] while PDE activity is linked to two structurally unrelated domains named EAL [26] and HD-GYP [27]. To prevent GTP wastage, DGC activity is under non-competitive product inhibition [28–29]. The primary inhibition site (Ip) is composed of a RxxD motif [30–31]. Bacterial responses to intracellular levels of c-di-GMP are mediated by c-di-GMP effectors. To date, two kinds of c-di-GMP effectors have been identified: c-di-GMP binding proteins and RNA riboswitches [17, 32]. Most of the c-di-GMP effector proteins control biofilm development by stimulating EPS biosynthesis [33–40] and inhibiting bacterial motility devices [41–45]. PilZ protein domains are widespread and are the main characterized c-di-GMP effector-domain family [33, 46–48]. Proteins with a PilZ domain regulate several phenotypes such as flagellar motility, twitching motility mediated by type IV pili and synthesis of EPS. Some c-di-GMP effector proteins with a degenerated GGDEF domain use the RxxD motif from the Ip to bind c-di-GMP [35, 49–50]. Conserved global bacterial regulators control the expression of many GGDEF, EAL, and HD-GYP domain-type genes in response to external signals, linking c-di-GMP signalling not only with motility and biofilm phenotypes, but also with bacterial metabolism [51].

A functional c-di-GMP pathway has been recently reported in *At*. *ferrooxidans* [52]. An increase in c-di-GMP levels resulted in *At*. *ferrooxidans* cells adhering to solid energy-providing substrates more effectively, indicating a role in biofilm formation and suggesting that the QS and the c-di-GMP pathways could be connected in this microorganism as it is in other Gram-negative bacteria [52].

Most of the data regarding molecular mechanisms involved in EPS production and biofilm formation by bioleaching bacteria are from just one mesophilic, iron- and sulfur- oxidizing bacterium, *At*. *ferrooxidans*. Sulfur oxidizing species such as *At*. *thiooxidans* and *At*. *caldus* are fundamental players within the bioleaching community. In addition, as a moderate thermophile, *At*. *caldus* is of special interest. As no genes involved in known QS systems have, however, been identified by analysing the *At*. *caldus* genome sequences [53], we focused on the characterization of the c-di-GMP pathway in this sulfur-oxidizing specie to develop a global view on biofilm formation by the *Acidithiobacillus* genus. We have previously suggested that *At*. *caldus* could have a functional c-di-GMP signalling pathway [54]. We report here the initial characterization of several *At*. *caldus* DGC enzymes, by heterologous complementation experiments in *Escherichia coli* and *Salmonella* Typhimurium. An *At*. *caldus* mutant strain lacking the DGC ACAty_C1319 was constructed and a comparative analysis of motility and adherence phenotypes of the mutant and wild type strains was carried out by motility assays on semi-solid medium and microscopy observations. In this work, we provide the first evidence that it is possible to genetically manipulate key bioleaching phenotypes such as motility in *At*. *caldus*.

## Materials and Methods

### Bacterial Strains, Plasmids and Primers

Bacterial strains, plasmids and primers used in this work are listed in Table 1. *At*. *caldus* ATCC 51756 was grown in mineral salt liquid medium (MSM) [6] at pH 4.5 when thiosulfate (0.5 mg/ml) or tetrathionate (2 mg/ml) was used as energy source, while the pH was 2.5 when elemental sulfur (1 mg/ml) was used. Cultures were incubated in shake flasks at 45°C and 100 rpm. Growth curves were performed by a daily cell counting using a Petroff-Hausser chamber. Solid medium was obtained by adding Phytagel (Sigma-Aldrich) 0.9% (wt/vol) in tetrathionate liquid medium. Kanamycin (150 μg/ml final concentration) was added to liquid and solid media when selection was required. The mating medium was obtained by adding yeast extract 0.05% (wt/vol) to solid medium [55].

**Table 1:** Bacterial strains, plasmids and primers used in this study.

	**Description**	**Referencee**
**Bacterial strains**		
*Acidithiobacillus caldus*		
ATCC 51756	Wild type	1
*Δc1319*	ATCC 51756 aca*ty*_C1319::Km^r^	This study
*Salmonella* Typhimurium		
UMR1	ATCC 14028–1 s Nal^r^	53
AdrA1f	UMR1 adrA101::MudJ	54
MAE282	UMR1 STM1703::Cm^r^	51
*Escherichia coli*		
MG1655	Standard reference strain F^−^, λ^−^, rph-1^a^	
AM70	MG1655 Δ*csgA*::cat^a^	50
LMG194	F^-^ Δ*lac*X74 *gal*E*thirps*LΔ*pho*A (Pvu II) Δ*ara*714 *leu*::Tn*10*	Invitrogen
HB101	F^-^ (*mcrC*-*mrr*) *hsdS20* (rB^-^mB^-^) *recA13 ara-14 proA2 lacY1*Δ^-^ *galK2 rpsL20*(Sm^r^) *Xyl-5 mtl-1 leuB6 thi-1 supE44*	58
**Plasmids**		
pBAD24	Arabinose-regulated expression vector, Amp^r^	55
pBAD24AdrA	pBAD24::*adrA*	This study
pBAD24_C1319	pBAD24::*acaty*_*c1319*	This study
pBAD24_C1325	pBAD24::*acaty*_*c1325*	This study
pBAD24_C1328	pBAD24::*acaty*_*c1328*	This study
pTOPO	Control vector allowing direct cloning of PCR products	Invitrogen
pTOPOAdrA_wt_	*adrA* gene cloned as PCR product into pTOPO vector	50
pTOPOYdaM	*ydaM* gene cloned as PCR product into pTOPO vector	50
pTOPOWspR	*wspR* gene cloned as PCR product into pTOPO vector	50
pTOPO_C1013	*acaty*_*c1013* gene cloned as PCR product into pTOPO vector	This study
pTOPO_C1184	*acaty*_*c1184* gene cloned as PCR product into pTOPO vector	This study
pTOPO_C1319	*acaty*_*c1319* gene cloned as PCR product into pTOPO vector	This study
pTOPO_C1325	*acaty*_*c1325* gene cloned as PCR product into pTOPO vector	This study
pTOPO_C1328	*acaty*_*c1328* gene cloned as PCR product into pTOPO vector	This study
pTOPO_C1360	*acaty*_*c1960* gene cloned as PCR product into pTOPO vector	This study
pGEM-T	Ap^r^; T-tailed PCR product cloning vector	Promega
pOT	Apr; the 557-bp origin of transfer region from pR388 cloned into the *Eco*RI site of pUC19	58
pOT_*acaty_c1319*	Ap^r^; A 2.2 kb fragment containing *acaty_c1319* cloned into *Xba*I site of pOT	This study
pOT_MUT_*acaty*_*c1319*::Km	Ap^r^Km^r^; the kanamycin resistance cassette from Tn*5* cloned into blunted, *Stu*I-*Bst*XI sites internal to *acaty*_*c1319* gene on pOT_ *acaty*_*c1319*	This study
pR388	TraWIncWTp^r^ Su^r^	58
**Primers**		
MUT_*acaty*_*c1319*_F	GCTCTAGAGCTTTCGCGATGGATACAGC	
MUT_*acaty*_*c1319*_R	GCTCTAGAGCGACGAGTTCAGACAGGGC	
*acaty*_*c1013*_F	ACCTCTAGATAAGGAGGCGCGCTGATGAACCTTGGCGGA	
*acaty*_*c1013*_R	ACCAAGCTTCA*ATGATGATGATGATGATGATGATG*GGGGTAACTGGGCAAGAAC	
*acaty*_*c1184*_F	ACCTCTAGATAAGGAGGGCCTGCGATGCGCAAATCCGAT	
*acaty*_*c1184*_R	ACCGTCGACCA*ATGATGATGATGATGATGATGATG*CGAGGGTAATCCTTGGGAA	
*acaty*_*c1319*_F	ACCTCTAGATAAGGAGGTTGGAACATGGTCCGAACCAAA	
*acaty*_*c1319*_R	ACCAAGCTTCA*ATGATGATGATGATGATGATGATG*GCCCATGGAATAGCTGACT	
*acaty*_*c1325*_F	ACCTCTAGATAAGGAGGTCTCGACATGCGCTCCGATTCC	
*acaty*_*c1325*_R	ACCAAGCTTCA*ATGATGATGATGATGATGATGATG*GCAAATGCTCATACACCAA	
*acaty*_*c1328*_F	ACCTCTAGATAAGGAGGGCAAAGGATGACCGAAGCTCAG	
*acaty*_*c1328*_R	ACCAAGCTTCA*ATGATGATGATGATGATGATGATG*AACATGATCTCGGATCGCT	
*acaty*_*c1960*_F	ACCGTCGACTAAGGAGGTGGGCGCATGGCCAACTGTCTG	
*acaty*_*c1960*_R	ACCAAGCTTCA*ATGATGATGATGATGATGATGATG*GCCATGGATGTCTAGATCT	

### Bioinformatic tools

Genome sequence and predicted proteome of *At*. *caldus* ATCC 23270 (GenBank CP005986.1, CP005989.1, CP005988.1, CP005987) was obtained from the NCBI website (http://www.ncbi.nlm.nih.gov). Hidden Markov models (HMMs) of GGDEF, EAL, HD, and PilZ domains were obtained from Trust Sanger Institute server, Pfam. Searches were performed by HMMER 2.3.2 (http://hmmer.janelia.org), using HMMs as input and the *At*. *caldus* ATCC 51756 proteome as the database. HD-GYP domains were finally defined by the identification of the GYP signature motif inside HD positive results. To search for PelD, FleQ and Clp homologues in *At*. *caldus*, amino acid sequences (NCBI accession N° AAG06449.1, AAY47667 and AAC37124.1) were used as query in a BlastP analysis (http://cmr.tigr.org/tigr-scripts/CMR/GenomePage.cgi). Sequence analysis was performed by using Artemis software (Sanger Institute Pathogen Sequencing Unit). Potential candidate proteins identified with Artemis were used to formulate a BlastP search of the non-redundant database at NCBI (www.ncbi.nlm.nih.gov). Further characterization of putative proteins was performed employing bioinformatic tools in the Expert Protein Analysis System portal (http://www.expasy.org/). Transmembrane domain predictions were done by SubCell (http://www.cbs.dtu.dk/services/SubCell/abstract.php).

### RNA extraction

*At*. *caldus* ATCC 51756 strain was grown in tetrathionate media as described above. Cells were harvested at late exponential phase and washed with acid water (pH 2.0). This was followed by alkaline lysis and RNA extraction by a modified hot-phenol method [56]. Briefly, two successive extractions with acid phenol (60°C), acid phenol/chloroform and chloroform were performed. RNA was precipitated overnight with 0.1 V of sodium acetate (3M) and 1 V of absolute ethanol. After centrifugation, RNA pellets were washed twice with ethanol at 70% and then treated with DNase I (Roche). Total RNA obtained was checked by 2% agarose gel electrophoresis and quantified by spectrometry.

### C-di-GMP extraction

Cells of *Salmonella* from 5 ml of overnight cultures were collected by centrifugation. Supernatants were removed, and cellular pellets were washed twice with sterile water. Bacterial cells were then inoculated in M9 medium using glycerol (1%) as an energetic source, and immediately induced with L- arabinose (0.2%) for 4 hours. Cells were collected by centrifugation and were resuspended in 0.7 ml of sonication buffer 50 mM Tris, pH 8.5. After sonication, cell debris were removed by centrifugation and c-di-GMP was extracted as described previously [57]. Supernatants were acidified with 0.35 ml of 1.2 M HClO_4_ (final concentration of 0.4 M) and then neutralized with 0.3 ml of 1.6 M K_2_CO_3_ on ice for 15 min before centrifugation at 12,000 × g for 10 min. New supernatants were filtered and concentrated at 60°C for 1 h by speedvac. For *At*. *caldus* cells, 400 ml of sulfur grown cultures were harvested by centrifugation and cells were washed twice with acidic water. Then, nucleotide extractions from *At*. *caldus* cells were performed as described above.

### C-di-GMP detection and quantification

In order to measure c-di-GMP levels in *S*. Typhimurium recombinant strains overexpressing *At*. *caldus* genes *acaty_c1319*, *acaty_c1325* and *acaty_c1328*, 20 μL of nucleotidic extracts were injected into an HPLC system equipped with a diode-array detector. A 15-cm Supelcosil LC-18-DB, 3 μm particle size, reversed phase column was used. Elution was at room temperature (22°C) with a flow rate of 0.5 ml/min, starting with 10 min in buffer A (120 mM KH_2_PO_4_, pH 6.0), followed by a linear gradient rising to 20% methanol in 10 min, ending with 10 min in buffer A. C-di-GMP was determined by co-elution and identical UV absorption spectra with a c-di-GMP standard (Biolog, Bremen, Germany). C-di-GMP concentration was calculated based on a calibration curve (y = 4.1204x, R^2^ = 0.967).

To increase the sensitivity of our measurements, nucleotidic extracts from *At*. *caldus* were analyzed by HPLC coupled to a mass spectrometer. Separation was performed on an Agilent 1290 HPLC System using a 2.0 × 50 mm Keystone Scientific Nucleosil C18 RP column (Sigma-Aldrich) at the Center for Metabolomics and Mass Spectrometry of The Scripps Research Institute, California, EE.UU. Briefly, nucleotidic extracts were dried by lyophilization and then solubilized in 10 mM ammonium acetate. HPLC separations were performed with a 10 mM ammonium acetate solution and a methanol gradient. The injection volume was 5 μL and the flow rate was 0.4 mL/min throughout the chromatographic run. The detection of c-di-GMP was performed on an Agilent 6490 triple quadrupole mass spectrometer equipped with an electro spray ionization source using selected reaction monitoring analysis in a positive ionization mode. Therefore, transitions m/z 691>152 and 691>135 were used for quantification and signal specificity, respectively.

### Molecular cloning of *At*. *caldus* genes encoding proteins with GGDEF domains

Genomic DNA from *At*. *caldus* strain ATCC 51756 was used as template for PCR experiments. The *acaty_c1013*, *acaty_c1184*, *acaty_c1319*, *acaty_c1325 acaty_c1328* and *acaty_c1960* genes encoding proteins with single DGC domains were amplified using specific primers containing restriction sites for cloning purposes (Table 1). The PCR products were separated by electrophoresis and directly purified from agarose gel with the specific Wizard kit (Promega), and cloned independently in pGEM-T plasmids (Promega). The recombinant plasmids were transformed into *E*. *coli* strain LMG194. The purified recombinant plasmids obtained by Miniprep Wizard kit (Promega) were digested with suitable restriction endonucleases. DNA-restriction fragments containing *At*. *caldus* genes were separated by electrophoresis, recovered from agarose gel and cloned into pTOPO (*acaty_c1013*, *acaty_c1184*, *acaty_c1319*, *acaty_c1325 acaty_c1328* and *acaty_c1960*) and pBAD24 (*acaty_c1319*, *acaty_c1325*, *acaty_c1328*) vectors. The pTOPO and pBAD24 recombinant plasmids harbouring *At*. *caldus* genes were transformed into the *E*. *coli* AM70 and *Salmonella enterica* serovar Typhimurium AdrA1f strains, respectively.

### Congo red binding assay

*S*. Typhimurium and *E*. *coli* cells were grown 48 h at 28°C on LB agar plates without salt and supplemented with Congo Red (CR) (40 μg/ml) and Coomassie brilliant blue G-250 (10 μg/ml). L(+)-arabinose (0.2%) and IPTG (1 mM) were used to overexpress *At*. *caldus* genes cloned in pBAD24 and pTOPO, respectively. Ampicillin (100 μg/ml), chloramphenicol (20 μg/ml) and kanamycin (30 μg/ml) were added to selective media.

### Construction of an *acaty_c1319* suicide plasmid

A suicide plasmid was constructed as previously described [55]. A 2.2 kb fragment containing the *acaty_c1319* gene was amplified by the PCR using specific primers with *Xba*I restriction sites. This fragment was pre-cloned in pGEM-T Easy (Promega) and excised by *Xba*I digestion. In parallel, the Ori-T containing vector pOT (56) was digested with *Xba*I. Digested pOT and *Xba*I-fragments were ligated by T4 ligase (Promega) to give pOT_*acaty_c1319*. This construct was digested with *Stu*I and *Bst*XI, removing almost the entire GGDEF domain, and the ends were filled in using T4 DNA polymerase (Fermentas). A 1.45 kb *Hin*dIII-*Sma* fragment from pSKM2 [55] carrying the kanamycin resistance cassette from Tn*5* was blunt-end cloned into the blunted sites of pOT_*acaty_c1319* to give pOT_MUT_*acaty_c1319*::Km. For conjugation experiments, pOT_MUT_*acaty_c1319*::Km was transformed into *E*. *coli* HB101 cells that contained pR388 by using kanamycin and trimethoprim resistance selection. Then, the mobilizable suicide vector was transferred from *E*. *coli* HB101-R388 to *At*. *caldus* by conjugation.

### Conjugation

*At*. *caldus* ATCC 51756 was cultured in thiosulfate liquid medium at 37°C for 5 days. *E*. *coli* HB101 cells were pre-adapted overnight in 50 ml of thiosulfate medium pH 4.5 supplemented with yeast extract 0.05% (wt/vol) at 37°C. Fifty ml of *E*. *coli* HB101 containing the suicide plasmid, and 500 ml of *At*. *caldus* were collected by centrifugation and washed twice with mineral salts medium (MSM). *E*. *coli* HB101 (pOT_MUT_*acaty_c1319*::Km) and *At*. *caldus* cells were resuspended separately in 250 μl of MSM and mixed 1:1. 100 μl of the mix was spread onto a Supor 0.2 μm filter (PALL Gelman Laboratory) and placed on the mating medium. After 5 days of incubation at 37°C, cells were harvested by scraping growth from the filters with a loop, washed twice in mineral salts solution and inoculated into 500 ml of thiosulfate liquid medium supplemented with kanamycin (150 μg/ml). Cultures were incubated at 37°C for seven days in shake flasks with aeration. Cells were diluted appropriately, plated on solid thiosulfate media with kanamycin (150 μg/ml), and cultivated for 6 days until the colonies appeared.

### Screening for single or double crossover mutants

To assess whether they were single or double crossovers, screening of transconjugants was done by colony and Southern blot hybridizations [55]. Kanamycin resistant colonies of *At*. *caldus* obtained from conjugation assays were replicated on selective solid media and incubated at 37°C for 6 days before blots were performed. Hybridization was under stringent conditions with a probe specific for the kanamycin resistance cassette [55]. Following this step, hybridized blots were stripped of the kanamycin probe and re-probed with a 520-bp *acaty_c1319* internal fragment (GGDEF probe). Both probes were labelled by PCR with digoxigenin according to the manufacturer’s recommendations (Roche). Signals obtained with both probes were considered as single-crossover mutants, while signals obtained only with the kanamycin probe corresponded to double-crossover mutants.

### Phenotypic assays in *At*. *caldus*

Wild type and Δ*c1319* strains of *At*. *caldus* were grown using sulfur coupons as an energy source. Attachment was evaluated indirectly by counting of planktonic cells. In parallel, sulfur coupons were fixed and prepared for scanning electron microscopy (SEM) and fluorescence microscopy (FM) as described by González et al. [13]. For FM visualizing, adhered cells were fixed in 4% p-formaldehyde and stained with 4,6-diamidino-2-phenylindole (DAPI) at 1 mg/mL. Finally, coupons were washed in sterile water, dried at room temperature and imaged. Two coupons and five fields per coupon were observed for each sample. To perform the motility assay, 10 μl of supernatant from cultures extracted during exponential phase were spotted on semi-solid media (phytagel 0.2%, pH 4.7 with tetrathionate as an energy source). Motility was evaluated by measuring the area occupied by bacterial cells after incubation for 7 days at 45°C.

### DNA manipulations and sequencing

Plasmid preparation, restriction endonuclease digestions, gel electrophoresis, ligations, and Southern blot/colony hybridizations were performed using standard methods [58]. All the plasmid constructs were checked by automatic DNA sequencing (MACROGEN, Korea).

## Results

### *In silico* identification of essential genetic components for a functional c-di-GMP signalling pathway in *At*. *caldus*

**Fig 1:**
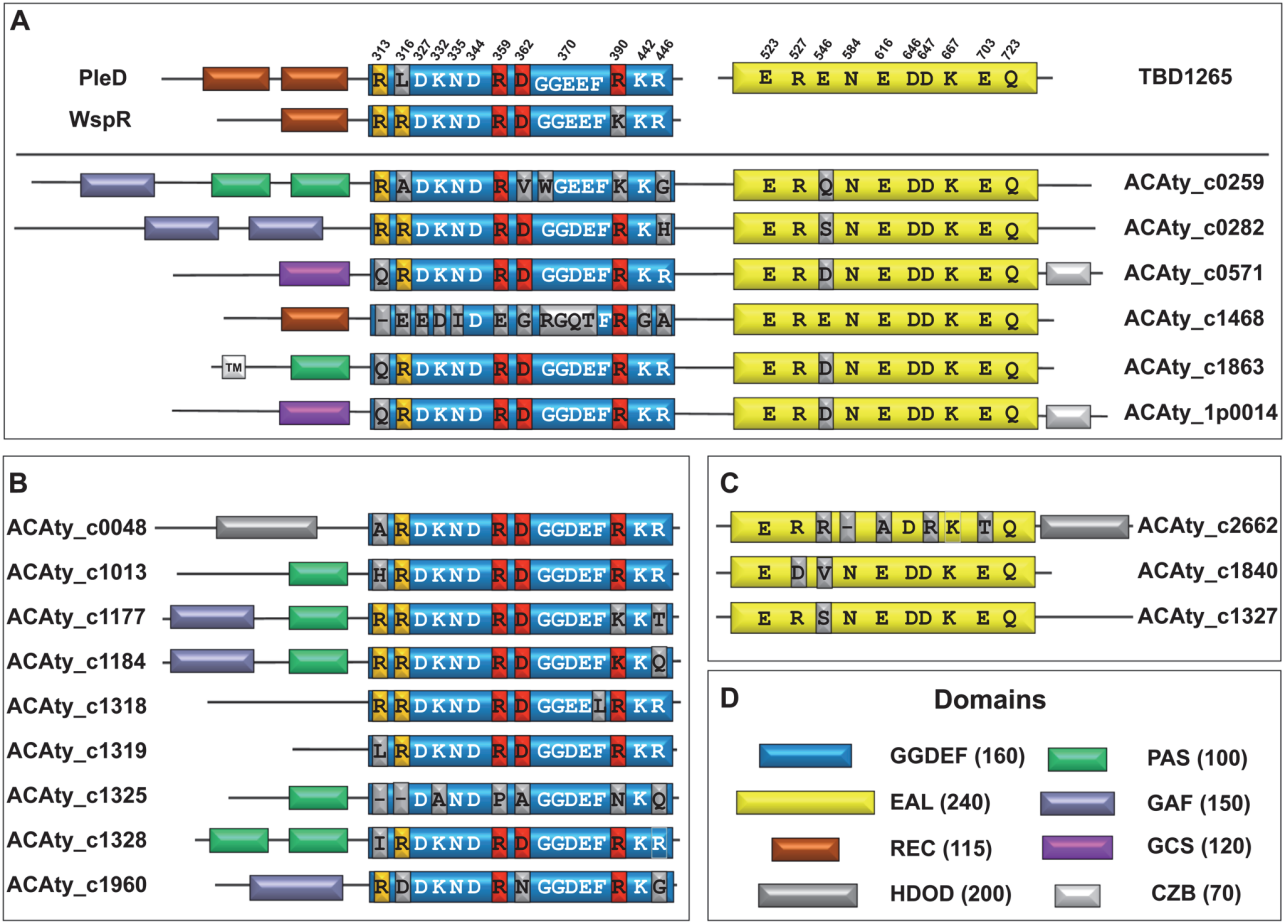
Domain organization and key amino acids of *At*. *caldus* proteins involved in c-di-GMP metabolism. Schematic representations of (**A**) GGDEF/EAL-, (**B**) single GGDEF- and (**C**) single EAL-domain containing proteins from *At*. *caldus* ATCC 51756. Color codes for the different domains are noted (**D**). Numbers in brackets indicate the average number of amino acids for each domain. GGDEF domains (blue) are compared to GGDEF domains of PleD and WspR from *C*. *crescentus* and *P*. *aeruginosa*, respectively (A, upper, left). EAL domains (yellow) are compared to the EAL domain of TBD1265 from *T*. *denitrificans* (A, upper, right). Numbers above the domains indicate key residues involved in DGC and PDE activities. Primary inhibition site (Ip) and secondary inhibition site (Is) residues of GGDEF domains are depicted in red and orange, respectively. Non-conserved amino acids are shown in gray. Domains are not drawn to scale. TM, transmembrane segment.

The bioinformatic analysis of the *At*. *caldus* ATCC 51756 genomic sequence allowed the identification of eighteen open reading frames (ORFs) related to c-di-GMP metabolism. Nine of them encode proteins with a single GGDEF domain (ACAty_c0048, ACAty_c1013, ACAty_c1177, ACAty_c1184, ACAty_c1318, ACAty_c1319, ACAty_c1325, ACAty_c1328, ACAty_c1960), three with a single EAL domain (ACAty_c1327, ACAty_c1840 y ACAty_c2662), and six with both GGEDEF and EAL domains (ACAty__1P_0014, ACAty_c0259, ACAty_c0282, ACAty_c0571, ACAty_c1468, ACAty_c1863) (Fig. 1). Sensor domains PAS (Period circadian protein, Aryl hydrocarbon receptor nuclear translocator protein and Single-minded protein), GAF (cGMP-specific phosphodiesterases, adenylyl cyclases and FhlA), REC (Response regulator receiver domain), Protoglobin and CZB (Chemoreceptor zinc-binding domain) have also been predicted for most of these proteins. All these sensor domains have been reported to play an important regulatory role in c-di-GMP signalling [25, 59–68]. No HD-GYP domains were identified from the *At*. *caldus* genome suggesting that PDE activity in *At*. *caldus* is likely to be generated by proteins containing only an EAL domain. On the other hand, nine ORFs encoding putative c-di-GMP effector proteins with a PilZ domain, and one encoding a PelD-like effector have been identified (Table 2). In addition, ACAty_c1318 could act as an effector protein since it possesses a conserved RxxD motif and an altered GGDEF motif (Fig. 1), where a phenylalanine substitution for leucine (GGDEF**→**GGDEL) should inhibit its DGC activity completely as occurs in the DGC WspR from *P*. *aeruginosa* [69]. Except for one of the PilZ effectors, in our experimental conditions RT-PCR assays revealed that all these c-di-GMP related ORFs were transcribed (S1 Fig.).

**Table 2:** C-di-GMP genes identified from available *Acidithiobacillus* genomes.

	*At. ferrooxidans*		*At. thioxidans*	*At. caldus*		*At. ferrivorans*
	ATCC 23270	ATCC 53993	ATCC 19377	ATCC 51756	SM-1	SS3
**GGDEF**	N.D.	1	9	9	8	3
**EAL**	1	1	3	3	3	N.D.
**GGDEF-EAL**	4	3	12	6	6	15
**HD-GYP**	N.D.	N.D.	1	N.D.	N.D.	1
**PilZ**	2	2	9^a^	9^a^	6^a^	6^a^
**PelD**	N.D.	N.D.	1	1	1	N.D.

N.D., Not Detected.

a, BcsA subunit of cellulose synthase is one of the PilZ domains.

### Detection of c-di-GMP in wild type strain ATCC 51756

To determine the presence of DGC activity, nucleotide extracts from *At*. *caldus* were analyzed by electrospray Ionization-Ion Trap mass spectrometry (ESI-IT). As described by Ruiz et al. (2012), negative polarity analysis allowed the identification of 344 m/z and 538 m/z MS/MS signals characteristic of c-di-GMP fragments (results not shown). This indicated that *At*. *caldus* possesses functional DGC activity as suggested by the previous identification of fifteen proteins with GGDEF domains.

### Analysis of the DGC activity of *At*. *caldus* proteins a with single GGDEF domain

**Fig 2:**
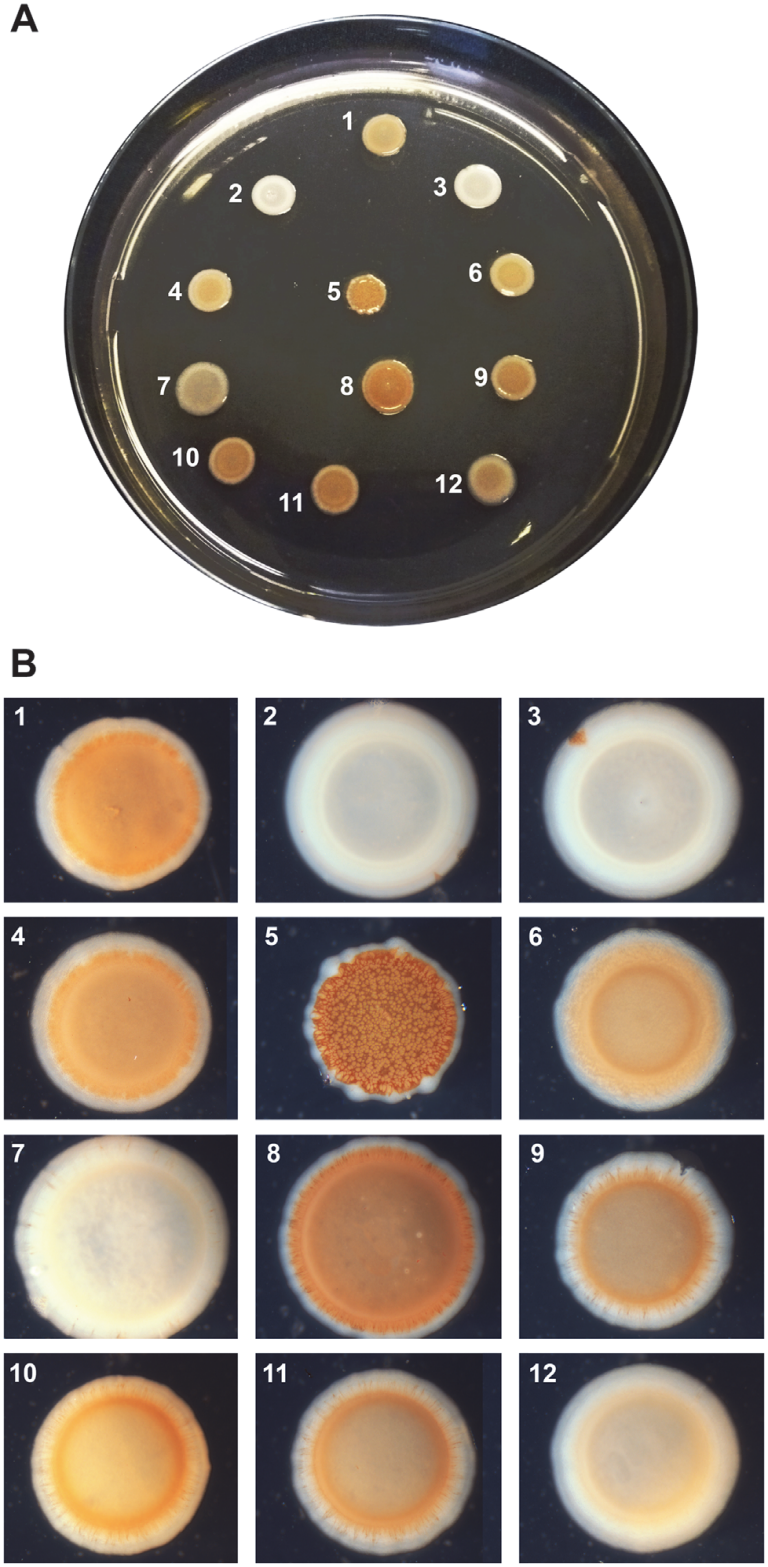
Heterologous complementation analysis in E. coli of the diguanylate cyclase activity from single GGDEF domains encoded by At. caldus genes. The ability of GGDEF-domain proteins to synthesize c-di-GMP and to induce a Red phenotype on a Congo red agar plate (**A**) by the *E*. *coli* MG1655 wild type strain (1), AM70 diguanylate cyclase null-mutant strain (2) and *E*. *coli* AM70 strains transformed with pTOPO2.1 vector carrying different *At*. *caldus* genes [*acaty_c1325* (7), *acaty_cC1319* (8), *acaty_c1328* (9), *acaty_c1960* (10), *acaty_c1184* (11), and *acaty_c1013* (12)] encoding GGDEF single domains were compared. A negative vector control without DNA insert (3) and three different diguanylate cyclase-carrying strains [YdaM (4), AdrA (5) or WspR (6)] were also analyzed. 10X magnification of each drop are shown in (**B**).

**Fig 3:**
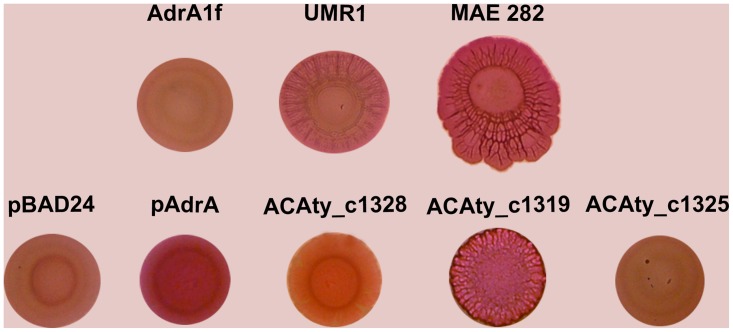
Heterologous complementation of Salmonella Typhimurium AdrAf1 strain by GGDEF-proteins ACAty_c1319, ACAty_c1325 and ACAty_c1328 from At. caldus. *S*. Typhimurium AdrA1f [Adr (DGC) null mutant] was complemented with pBAD24 plasmids harboring *At*. *caldus* genes coding for ACAty_c1319, ACAty_c1325 and ACAty_c1328 proteins. rdar morphotype was analyzed on Congo red agar plates and compared to wild type (UMR1), positive control (pAdrA), negative control (pBAD24 without insert) and a phosphodiesterase null mutant (MAE 282) strains.

It is well established that production of curli fibers and cellulose depends on c-di-GMP biosynthesis mediated by specific DGCs YdaM (curli fibers) in *E*. *coli* [70–71] and AdrA (cellulose) in *S*. Typhimurium [72–73]. Based on the presence of key amino acids [29, 31, 69], eleven GGDEF domains (ACAty__1P_0014, ACAty_c0048, ACAty_c0282, ACAty_c0571, ACAty_c1013, ACAty_c1177, ACAty_c1184, ACAty_c1319, ACAty_c1328, ACAty_c1863, ACAty_c1960) from *At*. *caldus* were predicted as active DGC while four (ACAty_c0259, ACAty_c1318, ACAty_c1325, ACAty_c1468) should be inactive DGC. To gain insight into c-di-GMP synthesis by *At*. *caldus*, six proteins with a single GGDEF domain (ACAty_c1013, ACAty_c1184, ACAty_c1319, ACAty_c1325, ACAty_c1328, ACAty_c1960) were assessed for DGC activity through Congo Red (CR) phenotype assays. CR is a diazo-dye with strong affinity for amyloid fibers, such as curli fibers produced by Enterobacteria and for polysaccharides such as cellulose. Genes *acaty_c1013*, *acaty_c1184*, *acaty_c1319*, *acaty_c1325*, *acaty_c1328* and *acaty_c1960*, were cloned and overexpressed in an *E*. *coli* AM70 strain. The different recombinant strains were tested for cellulose-production phenotype (Fig. 2). Positive results were obtained for *acaty_c1184*, *acaty_c1319*, *acaty_c1328* and *acaty_c1960* genes indicating that they code for functional DGC enzymes. An intermediate result was obtained with the *acaty_c1013* gene and the *E*. *coli* reporter system. Under our experimental conditions, no significant change was observed in the *E*. *coli* recombinant strains harbouring *At*. *caldus* gene *acaty_c1325* compared to the vector control without insert. This is in agreement with bioinformatic predictions that revealed the loss of two key amino acids involved in catalysis and GTP binding in the GGDEF domains of ACAty_c1325. The c-di-GMP signalling pathway has been associated with specific and compartmentalized metabolic activity [15, 74, 75]. To exclude the possibility that the negative result obtained with *acaty_c1325* was due to the reporter system, *acaty_c1325* was also cloned and overexpressed in *S*. Typhimurium AdrA1f (Fig. 3) to assess the multicellular red, dry and rough (rdar) morphotype which is also expressed upon the presence of a DGC activity [76]. In addition, to compare to the result obtained in the *E*. *coli* reporter system, *S*. Typhimurium recombinant strains harbouring *acaty_c1319* and *acaty_c1328* genes were also constructed (Fig. 3). In agreement with the results obtained with *E*. *coli*, recombinant strains *S*. Typhimurium *acaty_c1325* also failed to restore rdar morphotype while *acaty_c1328* and *acaty_c1319* genes did so partially and completely, respectively. Similar results were confirmed in calcofluor (CF) binding assays, which monitors the expression of cellulose (S2 Fig.). Altogether, these results indicate that *acaty_c1325* codes for an inactive DGC. As neither partner domains nor inhibition sites were identified in the GGDEF domain of ACAty_c1325, its function is still uncertain. Recently, the crystal structure of XCC4471^GGDEF^ from *X*. *campestris* showed that a dimer of GGDEF domains was joined through c-di-GMP dimer molecules located at the active sites [77]. The GGDEF domain of ACAty_c1325 could therefore still play a functional role as a c-di-GMP effector domain.

### Construction of a Δ*c1319* null mutant

**Fig 4:**
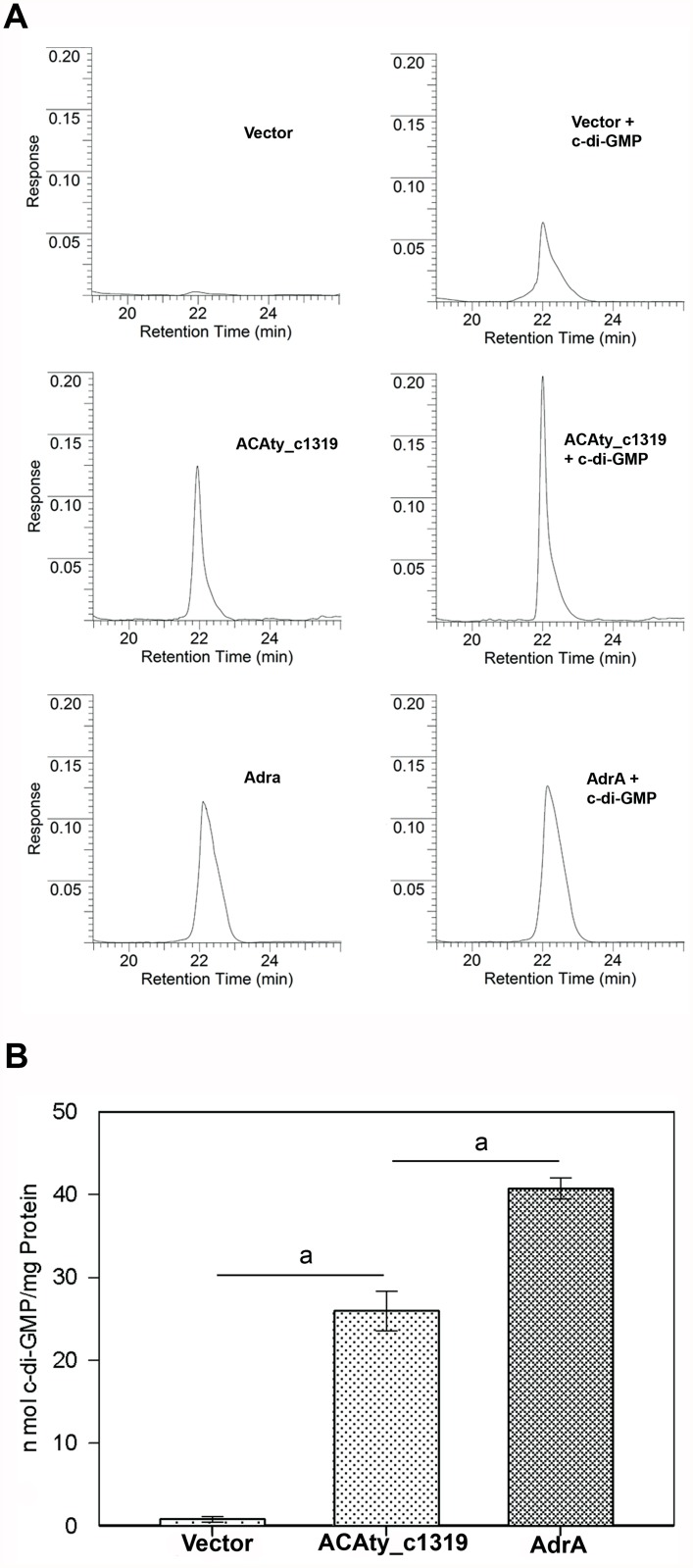
ACAty_c1319 is a functional diguanylate cyclase (DGC) enzyme. **A**. Cell extracts from recombinant strains of *S*. Typhimurium AdrA1f transformed with the pBAD24 plasmid vector without DNA insert, or harboring *At*. *caldus acaty_c1319* or *adrA* genes were supplemented with 2 nmol of synthetic c-di-GMP (**right panel**) or not *(* **left panel**) and analyzed by HPLC. **B**. HPLC quantifications of c-di-GMP intracellular levels. Values with standard deviations were obtained from triplicates. a, Statistical significance was determined by ANOVA (p < 0.05).

In both reporter systems, the highest positive results were obtained with the *acaty_c1319* gene (Fig. 4), indicating that this may encode a key DGC enzyme for *At*. *caldus*. No genetic transfert techniques are currently available in *Acidithiobacillus* sp. However, two recent reports described the construction of a specific null mutant gene in *At*. *caldus* [55, 78]. To assess whether this DGC enzyme is related to a specific phenotype in *At*. *caldus*, the construction of a *At*. *caldus* Δ*c1319* null mutant strain was challenged by using a marker exchange strategy as described by Van Zyl et al. [55]. Recombinant colonies were checked for single or double crossover events (S3 Fig., A) by colony blot hybridization (S3 Fig., B). From eighty colonies giving a positive result with the specific kanamycin probe, twenty-one gave a negative result with the specific internal GGDEF probe (S3 Fig., B) suggesting that these colonies were true double-crossover mutants. To confirm the correct location of the DNA exchange, Southern blot and PCR analysis were performed using genomic DNA from wild type as well as two potential double-crossover *At*. *caldus* clones (called Δ*c1319*_6 and Δ*c1319*_10) and the specific kanamycin probe (S3 Fig., C). Two specific restriction enzyme-digested DNA fragments (7.2 kb and 8.0 kb for *Nhe*I and *Eco*RI, respectively) corresponding to a double-crossover event were obtained for both mutated clones, while a negative result was observed with DNA from the wild type strain (S3 Fig., A and C). In addition, due to the replacement of a small piece of the *acaty_c1319* gene by the kanamycin resistance gene, PCR allowed the amplification of 2.8 kb DNA fragments from both mutated clones compared to the 1.8 kb DNA-fragment obtained from the wild type strain (not shown).

### **Comparative analysis of wild type and** Δ**c1319 strains**

**Growth**. No differences were observed when comparing the growth curves of wild type and both Δc1319 mutants in tetrathionate liquid media during 4 days (not shown).

**Fig 5:**
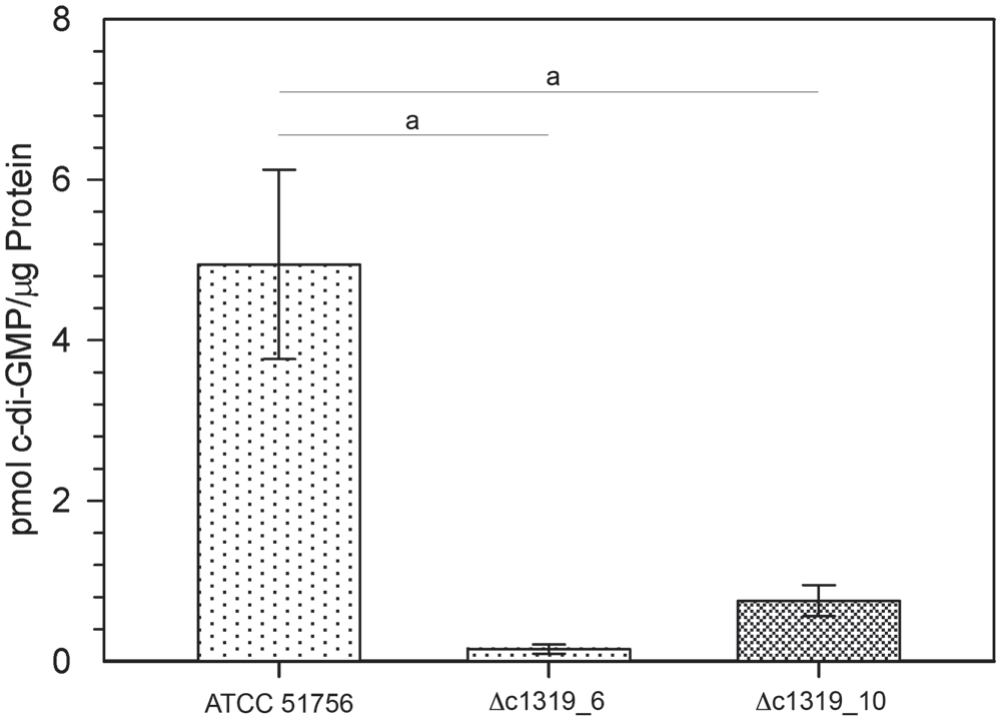
HPLC-MS/MS quantification of c-di-GMP intracellular levels in the *At*. *caldus* Δc1319 mutants. Values with standard deviations were obtained from triplicates. a, Statistical significance was determined by ANOVA (p < 0.05).

**C-di-GMP levels**. The levels of c-di-GMP were decreased by 13.75-fold in the Δc1319_6 mutant (0,36 ± 0,10 pmol of c-di-GMP per mg protein) compared to the wild type strain (4,95 ± 2,05 pmol of c-di-GMP per mg protein) as indicated in Fig. 5. A similar decrease (6.6-fold) was observed with the Δc1319_10 mutant. Thus, in our experimental conditions the DGC ACAty_c1319 was responsible for 85% to 93% of total c-di-GMP.

**Fig 6:**
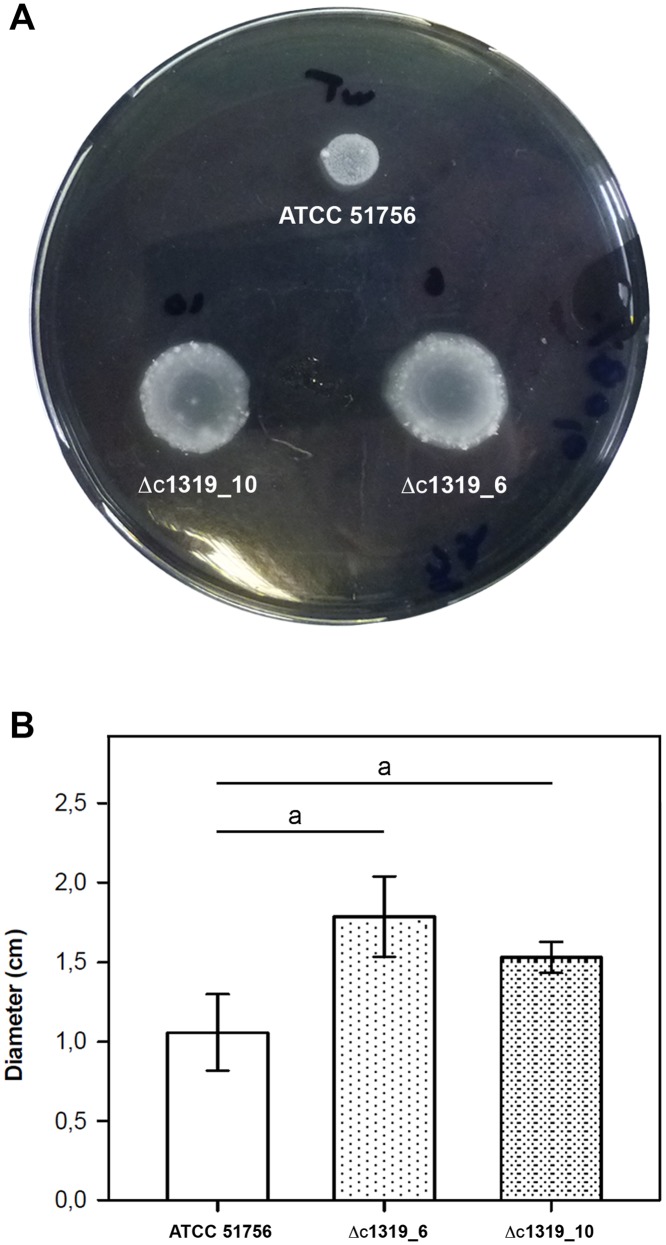
C-di-GMP modulates motility behavior in *At*. *caldus*. **A**, Motility behavior of *At*. *caldus* Δc1319 mutants on tetrathionate plates compared with wild type strain. **B**, Diameters of motility zones of *At*. *caldus* wild type ATCC 51756 and mutant Δc1319 strains after 96 h of incubation at 45°C. The error bars indicate standard deviations obtained from three independent trials. a, Statistical significance was determined by ANOVA (p < 0.05).

**Motility**. As no motility assays have been previously described for any *Acidithiobacillus* species, a specific semi-solid medium including phytagel and tetrathionate as gelling agent and energy source, respectively, was developed to analyse *At*. *caldus* motility. Results clearly indicated that the Δc1319 mutants have a higher motility than the wild type strain (Fig. 6).

**Fig 7:**
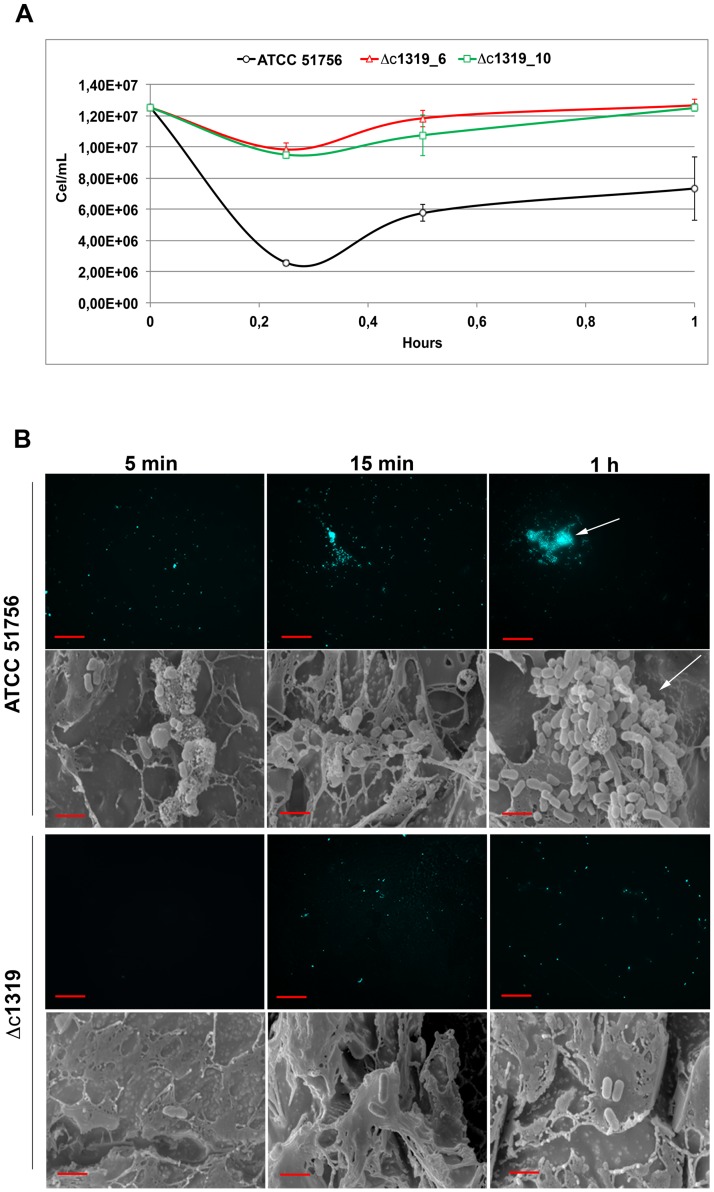
The ability to attach on sulfur coupons by At. caldus cells is decreased in Δc1319 mutant strains. **A**. Indirect determination of cell attachment by counting of remaining planktonic cells. **B**. Microscopy analysis of cell attachment on sulfur coupons by *At*. *caldus* wild type (upper) and Δc1319 mutant (lower). Sulfur coupons were retrieved from cultures at different times and visualized by scanning electron microscopy (SEM; 20 000X) and fluorescence microscopy (FM; 400X). As described by González et al. [13], cells attached on sulfur coupons were previously stained with 4′,6-diamidino-2-phenylindole (DAPI) for FM visualizing. Microcolonies are indicated by white arrows. Size bars represent 10 μM (FM) and 1 μM (SEM).

**Attachment to sulfur coupons**. Direct cell counting of liquid supernatants from cultures with elemental sulfur as energy source revealed that Δc1319_6 and Δc1319_10 planktonic cell numbers were higher compared with the wild type strain (Fig. 7A). To assess if these planktonic-cell number differences were related to the ability to attach on sulfur coupons, fluorescence and scanning electron microscopy analyses were performed (Fig. 7B). Similar results were obtained with both Δc1319 mutants. Both microscopy analyses clearly revealed that wild type strain (Fig. 7B, upper) was capable to develop microcolonies on sulfur coupons while it was possible to image only few isolated cells of mutant strains (Fig. 7B, lower). These results strongly suggested that the ability to attach on sulfur coupons by Δc1319 cells is lower compared with wild type cells.

## Discussion

In this study a bioinformatic, biochemical and genetic characterization of the c-di-GMP pathway of the acidophile *At*. *caldus* has been carried out. This is the first time that a DGC mutant strain of a bioleaching bacterium has been made and analysed.

An understanding of the number of genes involved in the c-di-GMP metabolism and their role in various environments faced by different bacteria is still lacking. In *Vibrio cholerae* in which c-di-GMP plays a key role in regulating changes in gene expression that occur during the shift from aquatic to host environments, 61 proteins (12 EAL, 30 GGDEF, 9 HD-GYP, and 10 GGDEF-EAL) related to c-di-GMP metabolism have been identified [79–80]. On the other extreme, *Mycobacterium smegmatis* has a single bifunctional protein with both GGDEF and EAL domains that plays a central role in long-term survival under conditions of nutritional starvation [81–82]. In this study, bioinformatic, biochemical and genetic strategies have been developed to characterize the c-di-GMP pathway of the acidophilic bacterium *At*. *caldus*. Eighteen ORFs (9 GGDEF, 3 GGDEF/EAL, 6 EAL) related to the metabolism of c-di-GMP have been identified. This number is higher than in *At*. *ferrooxidans* ATCC 23270 in which only five ORFs have been identified [52]. As a comparative analysis of genome sequence was aimed at predicting behavioural properties [83], we also searched for GGDEF and EAL domain proteins in other available genome sequences from *Acidithiobacillus* species (*At*. *caldus* SM-1, *At*. *ferrooxidans* ATCC 53993, *At*. *thiooxidans* ATCC 19377, *At*. *ferrivorans*). Our results revealed that the c-di-GMP metabolism pattern differs not only among species but also between strains from the same specie (Table 2). These results suggested that the complexity of c-di-GMP metabolism cannot be directly associated with the acidophilic ecological niche. However, proteins with single GGDEF and EAL domains and the PelD effector (Table 2) suggest that the global organization of the c-di-GMP pathway in sulfur-oxidizing *Acidithiobacillus* species is different to iron-sulphur species. Interestingly, in the course of identification of molecular mechanisms involved in biofilm formation in bioleaching bacterial species a canonical quorum sensing (QS) system has been identified in the iron-sulfur oxidizing specie *At*. *ferrooxidans* [11] while in both sulfur-oxidizing bacteria, specific genes related to QS are absent [53].

As a chemolithoautotrophic bacterium, *At*. *caldus* may require large quantities of energy to support carbon fixation, whereas molecular oxygen is the most suitable electron acceptor [84]. As found in other Gram-negative bacteria [78], in addition to their DGC and PDE domains, *At*. *caldus* enzymes involved in c-di-GMP metabolism possess sensor domains located in their N-terminal regions (Fig. 1). Interestingly most of them are PAS, GAF, and Protoglobin domains that are related to oxygen sensing and have been reported to regulate DGC or PDE activity in other bacteria [59–62, 65, 67, 68]. Hence, molecular oxygen availability may be an important stimulus to control intracellular levels of c-di-GMP in *At*. *caldus*.

Comparative assays between the wild type strain and the Δc1319 strain revealed that the presence of DGC ACAty_c1319 inhibits flagellar motility and is required for attachment on solid substrates (Figs. 6, 7). In the course of attempting to understand how both phenotypes could be regulated, several key c-di-GMP effectors have been singled out. Nine genes encoding proteins containing PilZ domains were identified in *At*. *caldus* ATCC 51756 (Table 2), seven of which have key amino acids for c-di-GMP binding. Among them, are four proteins related to type IV pili (T4P) assembly and one with significant similarity (27%) to YcgR. T4P has been shown to be involved in twitching motility in several bacterial species [85] whereas the binding of c-di-GMP by the YcgR protein family down-regulates the flagellar motor through interactions with flagellum components MotA and FliL located in the inner membrane of different Gram-negative bacteria [42–45]. Thus the location of orthologous flagellar genes *fliL*, *motA*, and *motB* upstream of *acaty_c1319* gene (S4 Fig.) strongly suggests that DGC ACAty_c1319 is involved in flagella functioning [44–45]. On the other side and immediately downstream of *acaty_c1319*, the *acaty_c1318* gene is predicted to encode a putative c-di-GMP effector protein with an inactive GGDEF domain. However, further studies are necessary to determine if FliL, MotAB, *ACAty_c1318* and *ACAty_c1319* are part of the same c-di-GMP regulatory module [15]. Regarding biofilm formation and EPS synthesis, orthologues of BcsA-BcsB (S5 Fig.) and PelD (S6 Fig.) effector proteins have been also identified. BcsA possesses a PilZ domain and as a subunit of the bacterial cellulose synthase [86] is responsible as a BcsA-BcsB complex for the synthesis of cellulose, an EPS frequently found in biofilms. PelD belongs to the Pel machinery and regulates PEL polysaccharide synthesis [35, 87]. As a complete *pel* operon including genes from *pelA to pelG* has been identified in *At*. *caldus* (S6 Fig.) and in this predicted PelD-like protein all of the key amino acids for c-di-GMP binding are conserved, we hypothesize that the synthesis of the Pel EPS occurs in *At*. *caldus* in a similar way that *P*. *aeruginosa*. In addition, a gene coding for an UDP-glucose 4-epimerase has also been identified at the 3’ end of this *pel* operon. This could be involved in the synthesis of PEL exopolysaccharide precursor. Moreover, it appears that such *pel*-like EPS operons are present only in the sulfur-oxidizing species *At*. *caldus* and *At*. *thiooxidans* while *bscAB* operon is present in both sulfur-oxidizing species and the iron-oxidizing species *At*. *ferrivorans* (Table 2).

Taken together these results demonstrate a specific role for the c-di-GMP pathway and Pel EPS for biofilm formation by a sulfur-oxidizing *Acidithiobacillus* species and comprise the first steps in deciphering the amazing cell network generated by bacterial acidophilic cells adhered to solid energetic substrate (S7 Fig.). However further genetic and biochemical studies are still necessary to understand if all these filaments protruding from cell bodies act cooperatively during cell attachment on solid energetic substrate and to determine if *At*. *caldus* is capable to produce specific bacterial adhesives such as the holdfast described in *Caulobacter crescentus* [88].

## Supporting Information

S1 Fig Expression analysis of *At*. *caldus* genes involved in c-di-GMP metabolism and signal transduction. Total RNA was extracted from *At*. *caldus* cells grown on elemental sulphur. All cDNA were synthesized from 1 μg of purified RNA by reverse transcriptase (+) and used as template for a conventional PCR with specific primers to amplify a 200–250 bp DNA fragment corresponding to the c-di-GMP synthesis (GGDEF), degradation (EAL) and signal transduction (PilZ and PelD) domains. (-) control without reverse transcriptase. The 200 bp fragment-size of DNA ladder is indicated by a red dot. The *acaty_c1319* gene encoding a DGC functional enzyme is indicated by a box.
(TIFF)

S2 Fig Heterologous complementation of S. Typhimurium AdrA1f strain by GGDEF-proteins ACAty_c1328, ACAty_c1319 and ACAty_c1325 from At. caldus. *S*. *typhimurium* AdrA1f [Adra (DGC) null mutant] was complemented with pBAD24 plasmids harboring *At*. *caldus* genes coding for ACAty_c1328, ACAty_c1319 and ACAty_c1325 GGDEF-proteins. Cellulose synthesis phenotype was analyzed by monitoring fluorescence intensity on Calcofluor (CF) agar plates compared to wild type (UMR1), positive control (pAdrA), negative control (pBAD24 without insert) and a phosphodiesterase null mutant (MAE 282) strains. The binding of CF to extracellular matrix was evident from the fluorescence intensity emitted under U.V. light.
(TIFF)

S3 Fig Confirmation of Δc1319 mutants by hybridization analysis. **A**, Schematic representation of the different possibilities for single and double recombination events of pOT_MUT_*aca_1319*::Kan suicide plasmid into the *At*. *caldus* genome. *Eco*RI and *Nhe*I restriction enzyme sites are given for comparison with results obtained by Southern blot analysis. Red boxes indicate locations of specific probes 1 and 2. **B**, Colony blot analysis. **C**, Southern blot analysis. Genomic DNA from wild type (WT) and Δ*1319_6* and Δ*1319_10* mutant strains of *At*. *caldus* was purified and digested by *Eco*RI and *Nhe*I. DNA fragments were separated by agarose gel electrophoresis and then transferred to a nitrocellulose membrane for hybridization with probe 2 (kanamycin) labelled with digoxigenin. Positive signals were obtained for clones 6 (lanes 5–7) and 10 (lanes 8–10) of Δ*1319* mutant strains while no signals were detected for WT strain (lanes 2–4). The sizes of positive *Eco*RI and *Nhe*I DNA fragments (lanes 6, 7, 9 and 10) correspond to double crossover events. DNA of the pOT_MUT_*acaty_c1319*:Kan plasmid employed for the mutagenesis was used as a positive control for hybridization (lanes 11 and 12). **ND**, not-digested. **MW**, Molecular weight.
(TIFF)

S4 Fig Genomic context of acaty_1319 gene from At. caldus ATCC 51756. Color code: gene coding for proteins with a predicted functional GGDEF (blue), EAL (yellow) and no functional GGDEF (cyan) domains and proteins involved in flagellar motility (green).
(TIFF)

S5 Fig Genomic organization of putative cellulose synthase operon from At. caldus ATCC 51756. Amino acid sequences of BcsA-BcsB subunits forming the catalytic core of cellulose synthase from *Glucoacetobacter xylinus* E25 and *At*. *caldus* have been compared. Percentage identity (I) and similarity (S) are noted. UGPU, UTP-glucose-1-phosphate uridylyltransferase.
(TIF)

S6 Fig Pel-like operon in At. caldus ATCC 51756. Amino acid sequences of the different proteins belonging to the *pel* operon from *At*. *caldus* and *P*. *aeruginosa* have been compared. Percentage identity (I) and similarity (S) are noted.
(TIF)

S7 Fig Biofilm network mediated by filament projections emerging from At. caldus cells grown on a sulfur coupon. Cells are able to directly contact other cells through filament projections that look like to holdfasts described in *Caulobacter crescentus* [88]. In several cases, connections appear to be achieved by more than one filament (blue arrows). Red arrows indicate putative sensing points between cooperative filaments from different cells and/or breaking points in several filaments. A putative holdfast-like extremity of a stalk protruding from the cell body is indicated by a yellow arrow.
(TIF)

## References

[1] Hallberg KB, Lindstrom EB (1994) Characterization of *Thiobacillus caldus* sp. nov., a moderately thermophilic acidophile. Microbiology 140: 3451–3456. 7533596 10.1099/13500872-140-12-3451

[2] Rawlings DE, Coram NJ, Gardner MN, Deane SM (1999) *Thiobacillus caldus* and *Leptospirillum ferrooxidans* are widely distributed in continuous- flow biooxidation tanks used to treat a variety of metal-containing ores and concentrates In Biohydrometallurgy and the environment toward the mining of the 21st century. Part A. Edited by: Amils R, Ballester A. Elsevier Press, Amsterdam, 777–786.

[3] Okibe N, Gericke M, Hallberg KB, Johnson DB (2003) Enumeration and characterization of acidophilic microorganisms isolated from a pilot plant stirred-tank bioleaching operation. Appl Environ Microbiol 69: 1936–1943. 12676667 10.1128/AEM.69.4.1936-1943.2003 PMC154788

[4] Foucher S, Battaglia-Brunet F, d’Hugues P, Clarens M, Godon JJ, et al (2003) Evolution of the bacterial population during the batch bioleaching of a cobaltiferous pyrite in a suspended-solids bubble column and comparison with a mechanically agitated reactor. Hydrometallurgy 71: 5–12.

[5] Dopson M, Lindstrom EB (2004) Analysis of community composition during moderately thermophilic bioleaching of pyrite, arsenical pyrite, and chalcopyrite. Microb Ecol 48:19–28. 15085303 10.1007/s00248-003-2028-1

[6] Dopson M, Lindstrom EB (1999) Potential role of *Thiobacillus caldus* in arsenopyrite bioleaching. Appl Environ Microbiol 65: 36–40. 9872756 10.1128/aem.65.1.36-40.1999 PMC90979

[7] Rohwerder T, Gehrke T, Kinzler K, Sand W (2003) Bioleaching review part A: progress in bioleaching: fundamentals and mechanisms of bacterial metal sulfide oxidation. Appl Microbiol Biotechnol 63: 239–248. 14566432 10.1007/s00253-003-1448-7

[8] Semenza M, Viera M, Curutchet G, Donati E (2002) The role of *Acidithiobacillus caldus* in the bioleaching of metal sulfides. Latin American applied research 32: 303–306.

[9] Edwards KJ, Bond PL, Banfield JF (2000) Characteristics of attachment and growth of *Thiobacillus caldus* on sulphide minerals: a chemotactic response to sulfur minerals? Environ Microbiol 2: 324–332. 11200434 10.1046/j.1462-2920.2000.00111.x

[10] Sand W, Gerke T (2006) Extracellular polymeric substances mediate bioleaching/biocorrosion via interfacial processes involving iron (III) ions and acidophilic bacteria. Res Microbiol 157: 49–56. 16431087 10.1016/j.resmic.2005.07.012

[11] Farah C, Vera M, Morin D, Haras D, Jerez CA, et al (2005) Evidence for a functional quorum-sensing type AI-1 system in the extremophilic bacterium *Acidithiobacillus ferrooxidans* . Appl Environ Microbiol 71: 7033–7040. 16269739 10.1128/AEM.71.11.7033-7040.2005 PMC1287726

[12] Rivas M, Seeger M, Holmes DS, Jedlicki E (2005) A Lux-like quorum sensing system in the extreme acidophile *Acidithiobacillus ferrooxidans* . Biol Res 38: 283–297. 16238107 10.4067/s0716-97602005000200018

[13] Gonzalez A, Bellenberg S, Mamani S, Ruiz L, Echeverria A, et al (2013) AHL signaling molecules with a large acyl chain enhance biofilm formation on sulfur and metal sulfides by the bioleaching bacterium *Acidithiobacillus ferrooxidans* . Appl Microbiol Biotechnol 97: 3729–3737. 10.1007/s00253-012-4229-3 22752316

[14] Ruiz LM, Valenzuela S, Castro M, Gonzalez A, Frezza M, et al (2008) AHL communication is a widespread phenomenon in biomining bacteria and seems to be involved in mineral-adhesion efficiency. Hydrometallurgy 94: 133–137.

[15] Hengge R (2009) Principles of c-di-GMP signalling in bacteria. Nat Rev Microbiol 7: 263–273. 10.1038/nrmicro2109 19287449

[16] Römling U, Simm R (2009) Prevailing concepts of c-di-GMP signaling. Contrib Microbiol 16: 161–181. 10.1159/000219379 19494585

[17] Sondermann H, Shikuma NJ, Yildiz FH (2012) You’ve come a long way: c-di-GMP signaling. Curr Opin Microbiol 15: 140–146. 10.1016/j.mib.2011.12.008 22226607 PMC3320698

[18] Mills E, Pultz IS, Kulasekara HD, Miller SI (2011) The bacterial second messenger c-di-GMP: mechanisms of signalling. Cell Microbiol 13: 1122–1129. 10.1111/j.1462-5822.2011.01619.x 21707905

[19] Römling U, Galperin M Y, Gomelsky M (2013) Cyclic di-GMP: the First 25 Years of a Universal Bacterial Second Messenger. Microbiol Mol Biol Rev 77: 1–52. 10.1128/MMBR.00043-12 23471616 PMC3591986

[20] Tischler AD, Camilli A (2004) Cyclic diguanylate (c-di-GMP) regulates *Vibrio cholerae* biofilm formation. Mol Microbiol 53: 857–869. 15255898 10.1111/j.1365-2958.2004.04155.x PMC2790424

[21] Simm R, Morr M, Kader A, Nimtz M, Römling U (2004) GGDEF and EAL domains inversely regulate cyclic di-GMP levels and transition from sessility to motility. Mol Microbiol 53: 1123–1134. 15306016 10.1111/j.1365-2958.2004.04206.x

[22] Abel S, Bucher T, Nicollier M, Hug I, Kaever V, et al (2013) Bi-modal distribution of the second messenger c-di-gmp controls cell fate and asymmetry during the *Caulobacter* cell cycle. PLoS Genet 9: e1003744 10.1371/journal.pgen.1003744 24039597 PMC3764195

[23] Schirmer T, Jenal U (2009) Structural and mechanistic determinants of c-di-GMP signalling. Nat Rev Microbiol 7: 724–735. 10.1038/nrmicro2203 19756011

[24] Ausmees N, Mayer R, Weinhouse H, Volman G, Amikam D et al (2001) Genetic data indicate that proteins containing the GGDEF domain possess diguanylate cyclase activity. FEMS Microbiol Lett 204: 163–167. 11682196 10.1111/j.1574-6968.2001.tb10880.x

[25] Ryjenkov DA, Tarutina M, Moskvin OV, Gomelsky M (2005) Cyclic diguanylate is a ubiquitous signaling molecule in Bacteria: Insights into Biochemistry of the GGDEF Protein Domain. J. Bacteriol. 187: 1792–1798. 15716451 10.1128/JB.187.5.1792-1798.2005 PMC1064016

[26] Schmidt AJ, Ryjenkov DA, Gomelsky M (2005) The ubiquitous protein domain EAL is a cyclic diguanylate-specific phosphodiesterase: enzymatically active and inactive EAL domains. J Bacteriol 187: 4774–4781. 15995192 10.1128/JB.187.14.4774-4781.2005 PMC1169503

[27] Ryan RP, Fouhy Y, Lucey JF, Crossman LC, Spiro S, et al (2006) Cell-cell signaling in *Xanthomonas campestris* involves an HD-GYP domain protein that functions in cyclic di-GMP turnover. Proc Natl Acad Sci U S A 103: 6712–6717. 16611728 10.1073/pnas.0600345103 PMC1458946

[28] Christen B, Christen M, Paul R, Schmid F, Folcher M, et al (2006) Allosteric control of cyclic di-GMP signaling. J Biol Chem 281 42: 32015–32024. 16923812 10.1074/jbc.M603589200

[29] De N, Navarro MV, Raghavan RV, Sondermann H (2009) Determinants for the activation and autoinhibition of the diguanylate cyclase response regulator WspR. J Mol Biol 393: 619–633. 10.1016/j.jmb.2009.08.030 19695263 PMC2760619

[30] Chan C, Paul R, Samoray D, Amiot NC, Giese B, et al (2004) Structural basis of activity and allosteric control of diguanylate cyclase. Proc Natl Acad Sci U S A 101 49: 17084–17089. 15569936 10.1073/pnas.0406134101 PMC535365

[31] Wassmann P, Chan C, Paul R, Beck A, Heerklotz H, et al (2007) Structure of BeF_3_-modified response regulator PleD: implications for diguanylate cyclase activation, catalysis, and feedback inhibition. Structure. 15: 915–927. 17697997 10.1016/j.str.2007.06.016

[32] Ryan RP, Tolker-Nielsen T, Dow JM (2012) When the PilZ don’t work: effectors for cyclic di-GMP action in bacteria. Trends in Microbiology 20: 235–242. 10.1016/j.tim.2012.02.008 22444828

[33] Pratt JT, Tamayo R, Tischler AD, Camilli A (2007) PilZ domain proteins bind cyclic diguanylate and regulate diverse processes in *Vibrio cholerae* . J Biol Chem 282: 12860–12870. 17307739 10.1074/jbc.M611593200 PMC2790426

[34] Merighi M, Lee VT, Hyodo M, Hayakawa Y Lory S (2007) The second messenger bis-(3′-5′)-cyclic-GMP and its PilZ domain-containing receptor Alg44 are required for alginate biosynthesis in *Pseudomonas aeruginosa* . Mol Microbiol 65: 876–895. 17645452 10.1111/j.1365-2958.2007.05817.x

[35] Lee VT, Matewish JM, Kessler JL, Hyodo M, Hayakawa Y, et al (2007) A cyclic-di-GMP receptor required for bacterial exopolysaccharide production. Mol Microbiol 65: 1474–1484. 17824927 10.1111/j.1365-2958.2007.05879.x PMC2170427

[36] Hickman JW, Harwood CS (2008) Identification of FleQ from *Pseudomonas aeruginosa* as a c-di-GMP-responsive transcription factor. Mol Microbiol 69: 376–389. 10.1111/j.1365-2958.2008.06281.x 18485075 PMC2612001

[37] Krasteva PV, Fong JC, Shikuma NJ, Beyhan S, Navarro MV, et al (2010) *Vibrio cholerae* VpsT regulates matrix production and motility by directly sensing cyclic di-GMP. Science 327: 866–868. 10.1126/science.1181185 20150502 PMC2828054

[38] Navarro MV, Newell PD, Krasteva PV, Chatterjee D, Madden DR, et al (2011) Structural basis for c-di-GMP-mediated inside-out signaling controlling periplasmic proteolysis. PLoS Biol 9: e1000588 10.1371/journal.pbio.1000588 21304926 PMC3032553

[39] Wilksch JJ, Yang J, Clements A, Gabbe JL, Short KR, et al (2011) MrkH, a novel c-di-GMP-dependent transcriptional activator, controls *Klebsiella pneumoniae* biofilm formation by regulating type 3 fimbriae expression. PLoS Pathog 7: e1002204 10.1371/journal.ppat.1002204 21901098 PMC3161979

[40] Fazli M, O’Connell A, Nilsson M, Niehaus K, Dow JM, et al (2011) The CRP/FNR family protein Bcam1349 is a c-di-GMP effector that regulates biofilm formation in the respiratory pathogen *Burkholderia cenocepacia* . Mol Microbiol 82: 327–341. 10.1111/j.1365-2958.2011.07814.x 21883527

[41] Wolfe AJ, Visick KL (2008) Get the message out: cyclic-Di-GMP regulates multiple levels of flagellum-based motility. J Bacteriol 190: 463–475. 17993515 10.1128/JB.01418-07 PMC2223684

[42] Paul K, Nieto V, Carlquist WC, Blair DF, Harshey RM (2010) The c-di-GMP binding protein YcgR controls flagellar motor direction and speed to affect chemotaxis by a ‘backstop brake’ mechanism. Mol Cell 38: 128–139. 10.1016/j.molcel.2010.03.001 20346719 PMC2929022

[43] Fang X, Gomelsky M (2010) A post-translational, c-di-GMP-dependent mechanism regulating flagellar motility. Mol Microbiol 76: 1295–1305. 10.1111/j.1365-2958.2010.07179.x 20444091

[44] Christen M, Christen B, Allan MG, Folcher M, Jeno P, et al (2007) DgrA is a member of a new family of cyclic diguanosine monophosphate receptors and controls flagellar motor function in *Caulobacter crescentus* . Proc Natl Acad Sci U S A 104: 4112–4117. 17360486 10.1073/pnas.0607738104 PMC1805490

[45] Boehm A, Kaiser M, Li H, Spangler C, Kasper CA, et al (2010) Second messenger-mediated adjustment of bacterial swimming velocity. Cell 141: 107–116. 10.1016/j.cell.2010.01.018 20303158

[46] Ryjenkov DA, Simm R, Römling U, Gomelsky M. (2006) The PilZ domain is a receptor for the second messenger c-di-GMP: the PilZ domain protein YcgR controls motility in enterobacteria. J Biol Chem 281: 30310–30314. 16920715 10.1074/jbc.C600179200

[47] Habazettl J, Allan MG, Jenal U, Grzesiek S (2011) Solution structure of the PilZ domain protein PA4608 complex with cyclic di-GMP identifies charge clustering as molecular readout. J Biol Chem 286: 14304–14314. 10.1074/jbc.M110.209007 21310957 PMC3077631

[48] Amikam D, Galperin MY (2006) PilZ domain is part of the bacterial c-di-GMP binding protein. Bioinformatics 22: 3–6. 16249258 10.1093/bioinformatics/bti739

[49] Duerig A, Abel S, Folcher M, Nicollier M, Schwede T, et al 2009 Second messenger-mediated spatiotemporal control of protein degradation regulates bacterial cell cycle progression. Genes Dev 23: 93–104. 10.1101/gad.502409 19136627 PMC2632171

[50] Abel S, Chien P, Wassmann P, Schirmer T, Kaever V, et al (2011) Regulatory cohesion of cell cycle and cell differentiation through interlinked phosphorylation and second messenger networks. Mol Cell 43: 550–560. 10.1016/j.molcel.2011.07.018 21855795 PMC3298681

[51] Jonas K, Melefors O, Römling U. (2009) Regulation of c-di-GMP metabolism in biofilms. Future Microbiol 4: 341–358. 10.2217/fmb.09.7 19327118

[52] Ruiz LM, Castro M, Barriga A, Jerez CA, Guiliani N (2012) The extremophile *Acidithiobacillus ferrooxidans* possesses a c-di-GMP signalling pathway that could play a significant role during bioleaching of minerals. Lett Appl Microbiol 54: 133–139. 10.1111/j.1472-765X.2011.03180.x 22098310

[53] Valdés J, Pedroso I, Quatrini R, Holmes DS (2008) Comparative genome analysis of *Acidithiobacillus ferrooxidans*, *A*. *thiooxidans* and *A*. *caldus*: Insights into their metabolism and ecophysiology. Hydrometallurgy 94: 180–184.

[54] Castro M, Ruíz LM, Barriga A, Jerez CA, Holmes D, Guiliani N (2009) c-di-GMP Pathway in Biomining Bacteria. Adv Mat Res 71–73: 223–226.

[55] van Zyl LJ, van Munster JM, Rawlings DE (2008) Construction of *arsB* and *tetH* mutants of the sulfur-oxidizing bacterium *Acidithiobacillus caldus* by marker exchange. Appl Environ Microbiol 74:5686–5694. 10.1128/AEM.01235-08 18658286 PMC2547032

[56] Guiliani N, Bengrine A, Borne F, Chippaux M, Bonnefoy V (1997) Alanyl-tRNA synthetase gene of the extreme acidophilic chemolithoautotrophic *Thiobacillus ferrooxidans* is highly homologous to *alaS* genes from all living kingdoms but cannot be transcribed from its promoter in *Escherichia coli* . Microbiology 143: 2179–2187. 9245807 10.1099/00221287-143-7-2179

[57] Antoniani D, Bocci P, Maciag A, Raffaelli N, Landini P (2010) Monitoring of diguanylate cyclase activity and of cyclic-di-GMP biosynthesis by whole-cell assays suitable for high-throughput screening of biofilm inhibitors. Appl Microbiol Biotechnol 85: 1095–1104. 10.1007/s00253-009-2199-x 19707751

[58] Sambrook J, Fritsch E and Manniatis F. 1989 Molecular cloning: a laboratory handbook. Cold Spring Harbour, N.Y.: Cold Spring Harbour Laboratory Press.

[59] Wan X, Tuckerman JR, Saito JA, Freitas TA, Newhouse JS, et al (2009) Globins synthesize the second messenger bis-(3′-5′)-cyclic diguanosine monophosphate in bacteria. J Mol Biol 388: 262–270. 10.1016/j.jmb.2009.03.015 19285985 PMC4301737

[60] Tuckerman JR, Gonzalez G, Sousa EH, Wan X, Saito JA, et al (2009) An oxygen-sensing diguanylate cyclase and phosphodiesterase couple for c-di-GMP control. Biochemistry 48: 9764–9774. 10.1021/bi901409g 19764732

[61] Qi Y, Rao F, Luo Z y Liang ZX (2009) A flavin cofactor-binding PAS domain regulates c-di-GMP synthesis in AxDGC2 from *Acetobacter xylinum* . Biochemistry 48: 10275–10285. 10.1021/bi901121w 19785462

[62] Kitanishi K, Kobayashi K, Kawamura Y, Ishigami I, Ogura T, et al (2010) Important roles of Tyr43 at the putative heme distal side in the oxygen recognition and stability of the Fe(II)-O2 complex of YddV, a globin-coupled heme-based oxygen sensor diguanylate cyclase. Biochemistry 49: 10381–10393. 10.1021/bi100733q 21067162

[63] Galperin MY (2010) Diversity of structure and function of response regulator output domains. Curr Opin Microbiol 13: 150–159. 10.1016/j.mib.2010.01.005 20226724 PMC3086695

[64] Draper J, Karplus K, Ottemann KM (2011) Identification of a Chemoreceptor Zinc-Binding Domain Common to Cytoplasmic Bacterial Chemoreceptors. J Bacteriol. 193: 4338–4345. 10.1128/JB.05140-11 21725005 PMC3165512

[65] Savakis P, De Causmaecker S, Angerer V, Ruppert U, Anders K, et al (2012) Light-induced alteration of c-di-GMP level controls motility of Synechocystis sp. PCC 6803. Mol Microbiol. 85: 239–51. 10.1111/j.1365-2958.2012.08106.x 22625406

[66] Chen MW, Kotaka M, Vonrhein C, Bricogne G, Rao F, et al (2012) Structural insights into the regulatory mechanism of the response regulator RocR from *Pseudomonas aeruginosa* in cyclic Di-GMP signaling. J Bacteriol. 194: 4837–4846. 10.1128/JB.00560-12 22753070 PMC3430337

[67] Chang AL, Tuckerman JR, Gonzalez G, Mayer R, Weinhouse H, et al (2001) Phosphodiesterase A1, a regulator of cellulose synthesis in *Acetobacter xylinum*, is a heme-based sensor. Biochemistry. 40: 3420–3426. 11297407 10.1021/bi0100236

[68] An S, Wu J, Zhang LH (2010) Modulation of *Pseudomonas aeruginosa* biofilm dispersal by a cyclic-Di-GMP phosphodiesterase with a putative hypoxia-sensing domain. Appl Environ Microbiol 76: 8160–8173. 10.1128/AEM.01233-10 20971871 PMC3008239

[69] Malone JG, Williams R, Christen M, Jenal U, Spiers AJ, et al (2007) The structure-function relationship of WspR, a *Pseudomonas fluorescens* response regulator with a GGDEF output domain. Microbiology 153:980–994. 17379708 10.1099/mic.0.2006/002824-0

[70] Weber H, Pesavento C, Possling A, Tischendorf G, Hengge R (2006) Cyclic-di-GMP-mediated signalling within the sigma network of *Escherichia coli* . Mol Microbiol 62: 1014–1034. 17010156 10.1111/j.1365-2958.2006.05440.x

[71] Lindenberg S, Klauck G, Pesavento C, Klauck E and Hengge R (2013) The EAL domain protein YciR acts as a trigger enzyme in a c-di-GMP signalling cascade in *E*. *coli* biofilm control. EMBO J. 32: 2001–2014. 10.1038/emboj.2013.120 23708798 PMC3715855

[72] Zogaj X, Nimtz M, Rohde M, Bokranz W, Römling U (2001) The multicellular morphotypes of *Salmonella typhimurium* and *Escherichia coli* produce cellulose as the second component of the extracellular matrix. Mol Microbiol 39: 1452–1463. 11260463 10.1046/j.1365-2958.2001.02337.x

[73] Kader A, Simm R, Gerstel U, Morr M and Römling U (2006) Hierarchical involvement of various GGDEF domain proteins in rdar morphotype development of *Salmonella enterica* serovar Typhimurium. Mol Microbiol. 60: 602–16. 16629664 10.1111/j.1365-2958.2006.05123.x

[74] Tuckerman JR, Gonzalez G, Gilles-Gonzalez MA (2011) Cyclic di-GMP activation of polynucleotide phosphorylase signal-dependent RNA processing. J Mol Biol 407: 633–639. 10.1016/j.jmb.2011.02.019 21320509

[75] Merritt JH, Ha DG, Cowles KN, Lu W, Morales DK, et al (2010) Specific control of *Pseudomonas aeruginosa* surface-associated behaviors by two c-di-GMP diguanylate cyclases. MBio 1: e00183–10. 10.1128/mBio.00183-10 20978535 PMC2957078

[76] Römling U (2005) Characterization of the rdar morphotype, a multicellular behaviour in Enterobacteriaceae. Cell. Mol. Life Sci. 62: 1234–1246 15818467 10.1007/s00018-005-4557-x PMC11139082

[77] Yang CY, Chin KH, Chuah ML, Liang ZX, Wang AH, et al (2011) The structure and inhibition of a GGDEF diguanylate cyclase complexed with (c-di-GMP)(2) at the active site. Acta Crystallogr D Biol Crystallogr 67: 997–1008. 10.1107/S090744491104039X 22120736

[78] Chen L, Ren Y, Lin J, Liu X, Pang X, et al (2012) *Acidithiobacillus caldus* sulfur oxidation model based on transcriptome analysis between the wild type and sulfur oxygenase reductase defective mutant. PLoS One 7: e39470 10.1371/journal.pone.0039470 22984393 PMC3440390

[79] Galperin MY, Nikolskaya AN, Koonin EV (2001) Novel domains of the prokaryotic two-component signal transduction systems. FEMS Microbiol Lett 203: 11–21. 11557134 10.1111/j.1574-6968.2001.tb10814.x

[80] Koestler BJ, Waters CM (2013) Exploring environmental control of cyclic di-GMP signaling in Vibrio cholerae by using the ex vivo lysate cyclic di-GMP assay (TELCA). Appl Environ Microbiol. 79: 5233–41. 10.1128/AEM.01596-13 23793642 PMC3753962

[81] Kumar M, Chatterji D (2008) Cyclic di-GMP: a second messenger required for long-term survival, but not for biofilm formation, in *Mycobacterium smegmatis* . Microbiology 154: 2942–2955. 10.1099/mic.0.2008/017806-0 18832301

[82] Bharati BK, Sharma IM, Kasetty S, Kumar M, Mukherjee R, et al (2012) A full-length bifunctional protein involved in c-di-GMP turnover is required for long-term survival under nutrient starvation in *Mycobacterium smegmatis* . Microbiology 158: 1415–1427. 10.1099/mic.0.053892-0 22343354

[83] Galperin MY, Higdon R, Kolker E (2010) Interplay of heritage and habitat in the distribution of bacterial signal transduction systems. Mol Biosyst. 6:721–728. 10.1039/b908047c 20237650 PMC3071642

[84] Rawlings DE (2005) Characteristics and adaptability of iron- and sulfur-oxidizing microorganisms used for the recovery of metals from minerals and their concentrates. Microbial Cell Factories. 4: 13 15877814 10.1186/1475-2859-4-13 PMC1142338

[85] Pelicic V (2014) Type IV pili: e pluribus unum? Mol Microbiol. 68: 827–37 10.1111/j.1365-2958.2008.06197.x 18399938

[86] Morgan JLW, McNamara JT, Zimmer J (2014) Mechanism of activation of bacterial cellulose synthase by cyclic di-GMP. Nature structural & molecular biology 21: 489–498. 10.1111/febs.13202 24704788 PMC4013215

[87] Franklin MJ, Nivens DE, Weadge JT, Howell PL (2011) Biosynthesis of the *Pseudomonas aeruginosa* extracellular polysaccharides, alginate, Pel, and Psl. Front Microbial 2: 167. 10.3389/fmicb.2011.00167 PMC3159412 21991261

[88] Berne C, Ma X, Licata NA, Neves BR, Setayeshgar S, et al (2013) Physiochemical properties of *Caulobacter crescentus* holdfast: a localized bacterial adhesive. J Phys Chem B. 117: 10492–503. 10.1021/jp405802e 23924278 PMC3926197

