# Supplementary figures and images for: Diguanylate Cyclase Null Mutant Reveals That C-Di-GMP Pathway Regulates the Motility and Adherence of the Extremophile Bacterium Acidithiobacillus caldus
Source: PLoS One. 2015 Feb 17;10(2):e0116399. doi: 10.1371/journal.pone.0116399 (PMC4331095; doi:10.1371/journal.pone.0116399)

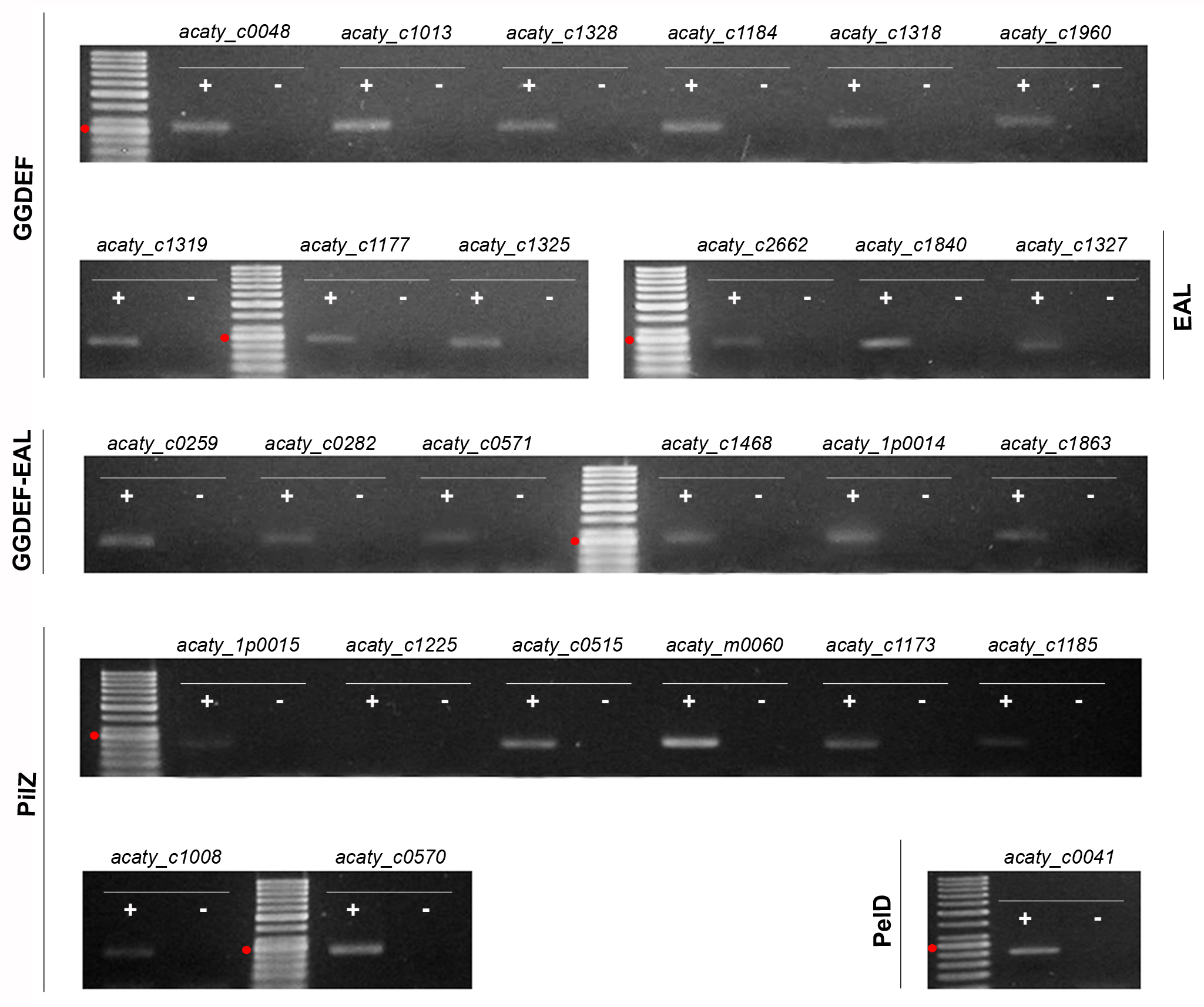

Supplement: S1 Fig — Total RNA was extracted from At. caldus cells grown on elemental sulphur. All cDNA were synthesized from 1 μg of purified RNA by reverse transcriptase (+) and used as template for a conventional PCR with specific primers to amplify a 200–250 bp DNA fragment corresponding to the c-di-GMP synthesis (GGDEF), degradation (EAL) and signal transduction (PilZ and PelD) domains. (-) control without reverse transcriptase. The 200 bp fragment-size of DNA ladder is indicated by a red dot. The acaty_c1319 gene encoding a DGC functional enzyme is indicated by a box. (TIFF) [file pone.0116399.s001.tiff]

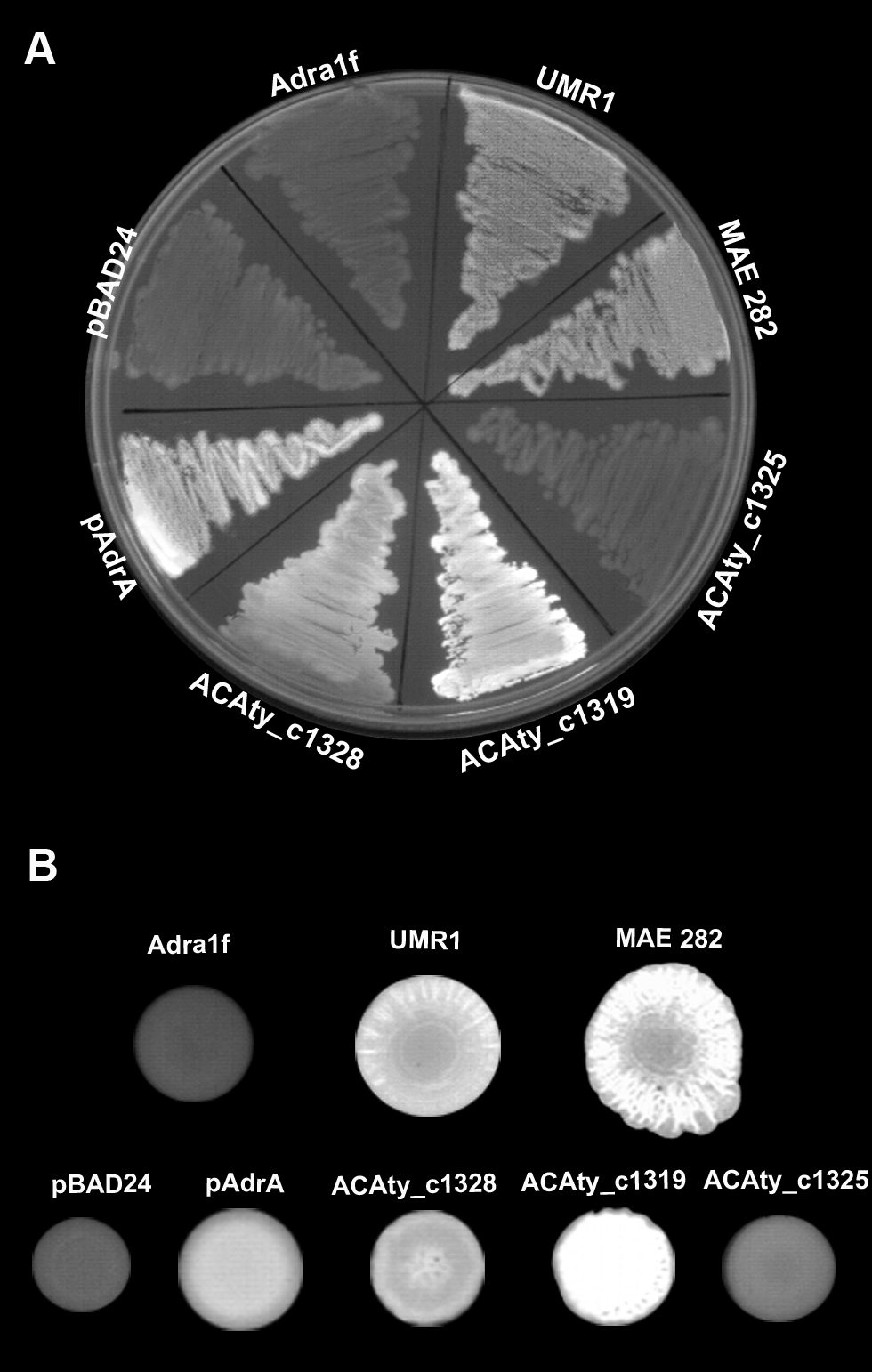

Supplement: S2 Fig — S. typhimurium AdrA1f [Adra (DGC) null mutant] was complemented with pBAD24 plasmids harboring At. caldus genes coding for ACAty_c1328, ACAty_c1319 and ACAty_c1325 GGDEF-proteins. Cellulose synthesis phenotype was analyzed by monitoring fluorescence intensity on Calcofluor (CF) agar plates compared to wild type (UMR1), positive control (pAdrA), negative control (pBAD24 without insert) and a phosphodiesterase null mutant (MAE 282) strains. The binding of CF to extracellular matrix was evident from the fluorescence intensity emitted under U.V. light. (TIFF) [file pone.0116399.s002.tiff]

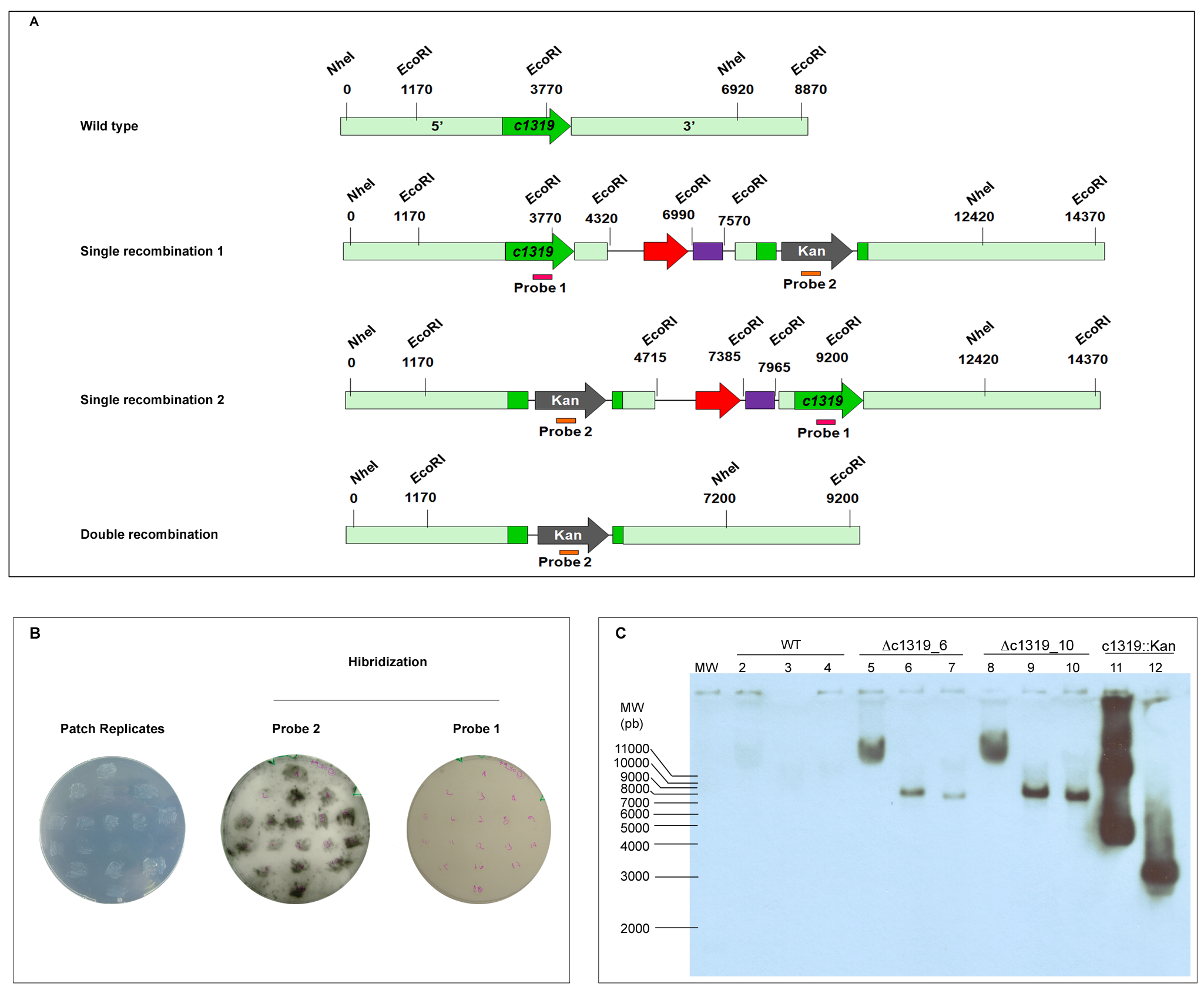

Supplement: S3 Fig — A, Schematic representation of the different possibilities for single and double recombination events of pOT_MUT_aca_1319::Kan suicide plasmid into the At. caldus genome. EcoRI and NheI restriction enzyme sites are given for comparison with results obtained by Southern blot analysis. Red boxes indicate locations of specific probes 1 and 2. B, Colony blot analysis. C, Southern blot analysis. Genomic DNA from wild type (WT) and Δ1319_6 and Δ1319_10 mutant strains of At. caldus was purified and digested by EcoRI and NheI. DNA fragments were separated by agarose gel electrophoresis and then transferred to a nitrocellulose membrane for hybridization with probe 2 (kanamycin) labelled with digoxigenin. Positive signals were obtained for clones 6 (lanes 5–7) and 10 (lanes 8–10) of Δ1319 mutant strains while no signals were detected for WT strain (lanes 2–4). The sizes of positive EcoRI and NheI DNA fragments (lanes 6, 7, 9 and 10) correspond to double crossover events. DNA of the pOT_MUT_acaty_c1319:Kan plasmid employed for the mutagenesis was used as a positive control for hybridization (lanes 11 and 12). ND, not-digested. MW, Molecular weight. (TIFF) [file pone.0116399.s003.tiff]

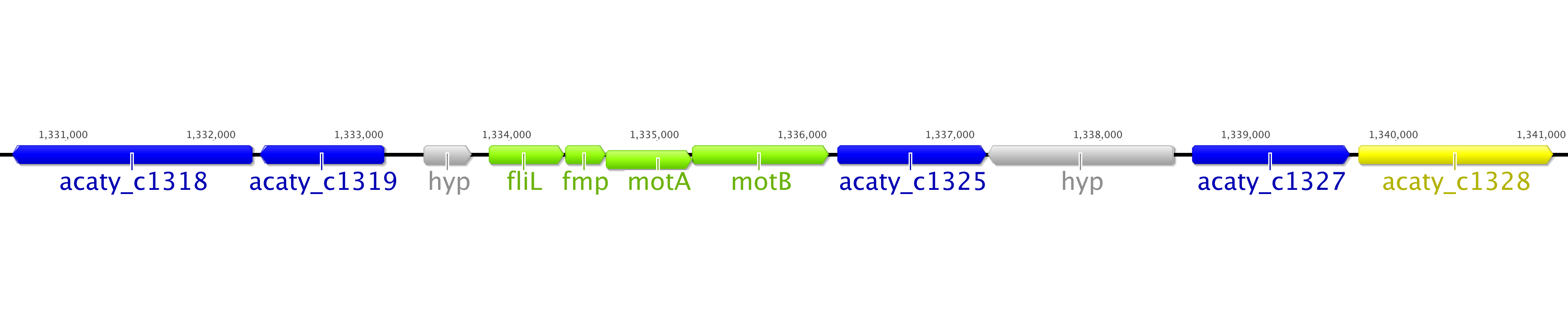

Supplement: S4 Fig — Color code: gene coding for proteins with a predicted functional GGDEF (blue), EAL (yellow) and no functional GGDEF (cyan) domains and proteins involved in flagellar motility (green). (TIFF) [file pone.0116399.s004.tiff]

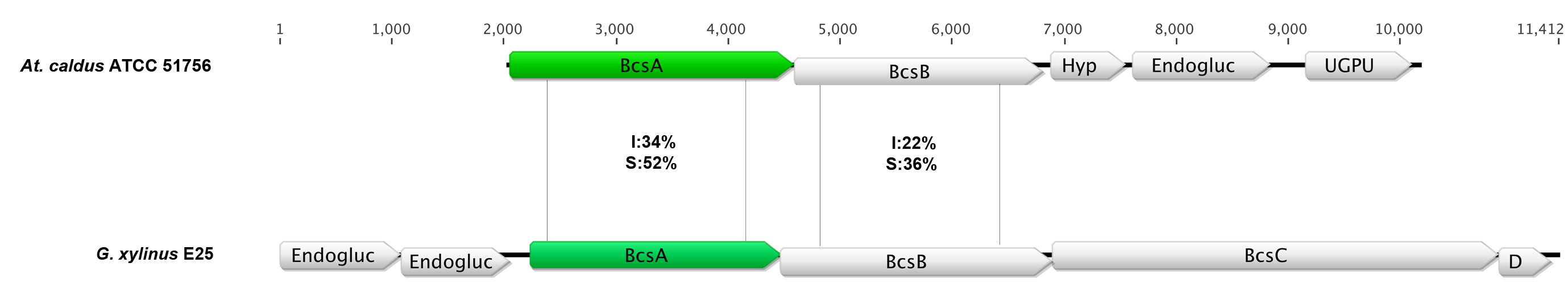

Supplement: S5 Fig — Amino acid sequences of BcsA-BcsB subunits forming the catalytic core of cellulose synthase from Glucoacetobacter xylinus E25 and At. caldus have been compared. Percentage identity (I) and similarity (S) are noted. UGPU, UTP-glucose-1-phosphate uridylyltransferase. (TIF) [file pone.0116399.s005.tif]

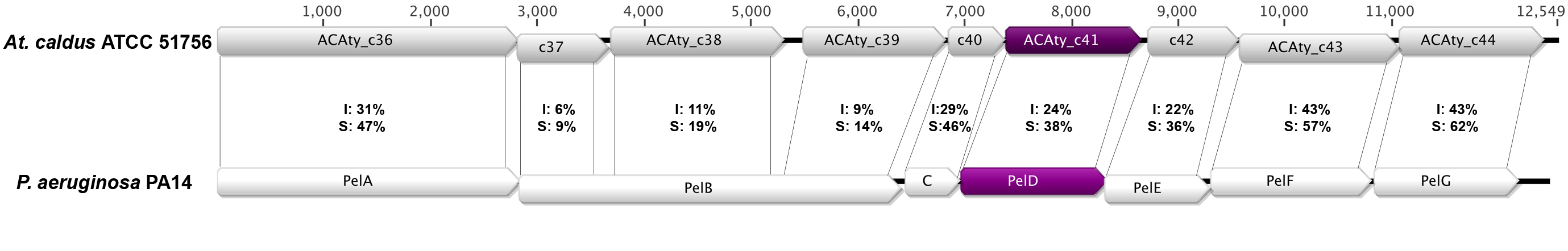

Supplement: S6 Fig — Amino acid sequences of the different proteins belonging to the pel operon from At. caldus and P. aeruginosa have been compared. Percentage identity (I) and similarity (S) are noted. (TIF) [file pone.0116399.s006.tif]

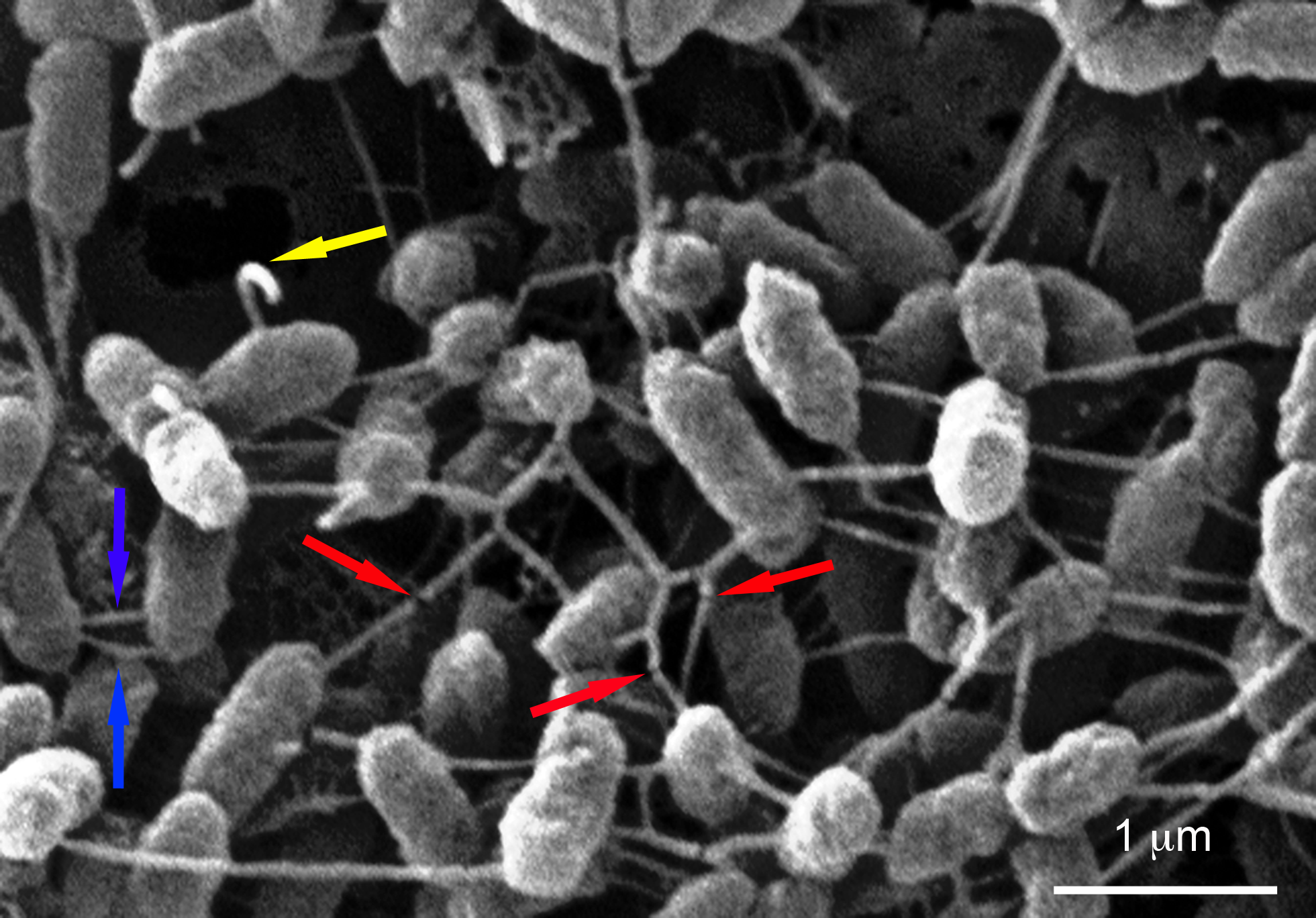

Supplement: S7 Fig — Cells are able to directly contact other cells through filament projections that look like to holdfasts described in Caulobacter crescentus [88]. In several cases, connections appear to be achieved by more than one filament (blue arrows). Red arrows indicate putative sensing points between cooperative filaments from different cells and/or breaking points in several filaments. A putative holdfast-like extremity of a stalk protruding from the cell body is indicated by a yellow arrow. (TIF) [file pone.0116399.s007.tif]
